# Effects of load-lightening and delayed extrapair benefits on the fitness consequences of helping behavior

**DOI:** 10.1093/beheco/arw018

**Published:** 2016-02-17

**Authors:** Caitlin A. Stern, Janis L. Dickinson

**Affiliations:** ^a^ Santa Fe Institute, 1399 Hyde Park Road, Santa Fe, NM 87501, USA,; ^b^ The Cornell Lab of Ornithology, Cornell University, 159 Sapsucker Woods Road, Ithaca, NY 14850, USA, and; ^c^ Department of Natural Resources, Cornell University, Fernow Hall, Ithaca, NY 14853, USA

**Keywords:** age-biased paternity, cooperative breeding, extrapair paternity, helping, inclusive fitness, indirect fitness benefits.

## Abstract

In most cooperative breeders, helping is directed at close kin, allowing helpers to gain indirect fitness benefits by increasing the reproductive success of close relatives, usually their parents. Extrapair paternity (EPP) occurs at high rates in some cooperative breeders, reducing the relatedness of helpers to the young they help raise. Even so, a son that helps is related to the brood by at least 0.25 through his mother and to within-pair young by 0.5, whereas a potential helper that has EPP in his own nest is related only to the offspring he sires and unrelated to any extrapair offspring. In birds, EPP often favors older males, which in the extreme case can result in sons being more closely related to young in their parents’ nest than to young in their own nests. The fitness benefit of helping will thus be enhanced if helping lightens the workload and increases survival of helpers and their fathers, enabling them to become old, hyper-successful extrapair sires. Here, we develop and analyze a proof-of-concept model, grounded in the western bluebird (*Sialia mexicana*) system, demonstrating the conditions under which high population levels of EPP can generate inclusive fitness benefits of helping behavior that outweigh the costs. This model provides a new perspective on the relationship between EPP and helping behavior in cooperative breeders and suggests a strong need for empirical work to gather unprecedented data on paternity over the lifetime of helpers and their parents.

## Lay Summary

Why do young animals forgo breeding to help raise their parents’ offspring, especially in birds, where a son’s relatedness to his parents’ young is often reduced by matings outside the pair-bond? If females’ extrapair partners are usually older males, and working less hard as a helper increases a son’s survival, EPP can enhance, rather than reduce, the benefits of helping. This new idea can help explain why species with helpers are often genetically nonmonogamous.

Twitter: @CaitlinStern

## INTRODUCTION

Cooperative breeding—characterized by joint care of young by 3 or more group members—is taxonomically widespread in animals, occurring at low frequencies in invertebrates, birds, and mammals (Brown 1987; Stacey and Koenig 1990; Solomon and French 1997; Bourke 2011). The predominant form of cooperative breeding involves pairs with helpers at the nest, typically offspring from prior broods. While most studies of cooperative breeding have demonstrated that helping is making the best of a bad job in the face of a shortage of resources or mating opportunities, these conclusions are rarely based on lifetime fitness measures that include genetic parentage (Dickinson and Hatchwell 2004; Koenig and Dickinson 2015). Here, we explore the novel hypothesis that age-dependent success at extrapair paternity (EPP) amplifies the advantages of helping, providing delayed direct and indirect fitness benefits, which are the 2 components of inclusive fitness (as articulated by Brown 1980). We refer to this new idea as the “delayed extrapair benefits” hypothesis.

Direct fitness benefits arise when helping increases personal reproduction, while indirect fitness benefits arise when helping increases the production of non-descendent kin (Brown 1980). EPP diminishes relatedness, *r*, between a helper and the young in his parents’ brood, which is often assumed to reduce the indirect fitness benefits of helping, *rB*, compared to the costs, *C*. Based on inclusive fitness theory, this would reduce the likelihood that Hamilton’s rule (*rB* > *C*) will be met (Hamilton 1964). This framing of the problem has led researchers to consider monogamy to be an important factor in the evolution of helping (the “monogamy hypothesis”; Boomsma 2007, 2009), and indeed this is straightforward for female helpers: a female that breeds independently is sure to be the mother of all the offspring in her brood (assuming an absence of brood parasitism), so a high probability that offspring in her parents’ brood are sired by an extrapair male would likely reduce the inclusive fitness benefits of helping relative to those of breeding. Phylogenetic studies support an association between cooperative breeding and monogamy, based on evidence that transitions to cooperative breeding are associated with reduced rates of EPP (Hughes et al. 2008; Cornwallis et al. 2010; Lukas and Clutton-Brock 2012). However, EPP ranges widely in cooperatively breeding birds from the Florida scrub-jay, *Aphelocoma coerulescens*, which is the most socially and genetically monogamous bird known (Quinn et al. 1999) to the superb fairy-wren, *Malurus cyaneus*, which is arguably the most genetically promiscuous (Double et al. 1997). This variation raises the question of why helping occurs in populations with high rates of EPP.

A recent critique of the monogamy hypothesis points to its failure to acknowledge that the indirect fitness benefits of helping depend on *relative*, not *absolute*, relatedness (Kramer and Russell 2014). Earlier, the idea that relative relatedness could favor helping was explicated formally for western bluebirds, *Sialia mexicana*, based on the idea that EPP not only reduces relatedness of male helpers to offspring in their parents’ nests (helper sons are related to extrapair half-sibs by 0.25) but also reduces relatedness of would-be helpers to extrapair young in their own nests by even more (e.g., to 0) such that sons could be more closely related to young in their parents’ nests than to young in their own nests (Dickinson et al. 1996, figure 6 within). This means that EPP can favor helping for sons with low paternity in their own nests. However, EPP is unlikely to favor helping for daughters, because an independently breeding female is (in the absence of brood parasitism) the genetic mother of all offspring in her nest. Indeed, in birds, females are less likely to help than are males (Brown 1987).

In addition to the possibility that a male in a high EPP population may be more closely related to half-siblings in his parents’ nest than to his own social offspring, EPP has the potential to favor helping behavior through increased survival and concomitant extrapair benefits that increase with the age of helpers or their parents. When these effects combine, it becomes clear that EPP can increase, rather than reduce, the relative benefits of helping for male helpers, especially when they are young. The delayed extrapair benefits hypothesis offers a novel explanation for why males help in species with high rates of EPP and prompts us to analyze under which conditions EPP can favor helping.

An advantage for older males in gaining EPP is widespread across bird species (Cleansby and Nakagawa 2012) and could affect the fitness consequences of helping behavior in 2 ways. First, a young male that delays breeding in order to help will suffer a lower direct fitness cost of helping than would an older male that helped; this is because a young male has an elevated chance of suffering EPP in his own nest and a reduced chance of gaining EPP as an independent breeder. Second, if helping positively affects the survivorship of the helper male and/or his father, both will have a higher likelihood of exploiting the advantage an extra year of age gives them in gaining EPP in the next breeding season. Of course, a positive effect on his mother’s survivorship will yield future indirect fitness benefits for the helper as well, but this effect occurs regardless of whether age influences EPP success for males. Helpers can increase their own probability of survival to the next breeding season, as well as that of the breeders they help, through load-lightening. Load-lightening occurs when the 2 parents and helper reduce their feeding effort below what they would invest as part of a pair breeding without a helper, such that each individual expends less energy on feeding young (Brown et al. 1978; Crick 1992; Hatchwell 1999). Thus, age-biased paternity can both reduce the direct fitness costs of helping and, in concert with load-lightening, increase the future direct and indirect fitness benefits of helping.

Here, we analyze a proof-of-concept model that demonstrates the conditions under which high population rates of EPP can result in increased inclusive fitness benefits of helping-at-the-nest. Proof-of-concept models are important in advancing understanding of evolution because, by formalizing a verbal hypothesis, they provide a rigorous test of the underlying logic (Servedio et al. 2014). We first build a model incorporating an age-dependent paternity bias favoring older males and ask whether this bias alone can result in larger inclusive fitness benefits from helping than from independent breeding; we find that this is the case under a small range of conditions. We then add to this basic model a reduction in annual mortality for adults in groups with a helper, including the helper, whose load is often lightened compared to if he bred independently. We find that adding load-lightening to the age-biased paternity model expands the range of conditions under which the benefits of helping can outweigh those of breeding, especially when male lifespans are long. Parameterizing our model with data on western bluebirds, we investigate this issue in a species with both helping behavior and a moderate incidence of EPP. Our model thus rigorously tests the verbal logic of the delayed extrapair benefits hypothesis.

## METHODS

### Age-biased paternity model

This model examines the factors that affect the inclusive fitness consequences of breeding versus helping where, in a given breeding season, a son’s options are either to breed independently or to help his social father and mother to raise their offspring. Although helpers can help a parent and step-parent, it is reasonable to consider the case of a helper assisting both parents. For example, in western bluebirds, sons are 6 times as likely to help both social parents than a parent and step-parent (Dickinson et al. 1996). In accordance with evidence suggesting that individuals can recognize social but not genetic kin, in western bluebirds as well as other bird species (Dickinson and Akre 1998; Dickinson 2004a; Greig et al. 2012; Griffin et al. 2013), we assume only recognition of social kin. All parameters are listed and defined in Table 1, and the logic of the inclusive fitness functions is shown in Table 2.

**Table 1:** The terms used in the models

Term	Definition	Range
*A*	Focal male’s age in the current breeding season	*A* ≥ 1
*d*	Age difference between the focal male and his father	*d* ≥ 1
*g*	Influence of age on success in gaining paternity (“age boost”)	0 < *g* ≤ 1
*s*	Relationship between probability of siring WPY versus EPY	0 ≤ *s* ≤ 1
*L*	Load-lightening effect on annual mortality	0 ≤ *L* ≤ 1
$p_{e}=1-\frac{g}{A}$	Focal male’s probability of siring EPY	
$p_{w}=1-\frac{sg}{A}$	Focal male’s probability of siring WPY	
*A* + *d*	Focal male’s father’s age in the current breeding season	
$p_{ef}=1-\frac{g}{A+d}$	Focal male’s father’s probability of siring EPY	
$p_{wf}=1-\frac{sg}{A+d}$	Focal male’s father’s probability of siring WPY	
$p_{wd}=1-\frac{sg}{d}$	Father’s probability of siring WPY the year the focal male was born	
*m*_*b*_	Annual mortality probability of an adult breeding in the absence of a helper	
*m*_*h*_ = *Lm*_*b*_	Annual mortality probability of an adult in a group that includes a helper	
*o*_*b*_	Number of offspring in a brood without a helper	
*o*_*h*_	Number of offspring in a brood with a helper	
*o*_*e*_	Number of EPY a male sires if he sires EPY	
*r*_*o*_	Relatedness of focal male to his genetic offspring	
*r*_*s*_	Relatedness of focal male to offspring of social father and mother	
*r*_*f*_	Relatedness of focal male to his father’s EPY	
*r*_*m*_	Relatedness of focal male to his mother’s EPY	

EPY, extrapair young; WPY, within-pair young.

**Table 2:** Inclusive fitness calculations

	Relatedness to offspring	Probability of offspring occurring	Number of offspring
Focal male breeds			
Direct fitness			
Focal male’s WPY	*r*_*o*_	$p_{w}=1-\frac{sg}{A}$	*o*_*b*_
Focal male’s EPY	*r*_*o*_	$p_{e}=1-\frac{g}{A}$	*o*_*e*_
Indirect fitness			
Social father’s EPY	*r*_*f*_	$p_{ef}=1-\frac{g}{A+d}$	*o*_*e*_
Mother’s EPY	*r*_*m*_	1 − *p*_*wf*_	*o*_*b*_
Social father and mother’s WPY	*r*_*s*_	$p_{wf}=1-\frac{sg}{A+d}$	*o*_*b*_
Inclusive fitness of breeding: *r*_*o*_(*p*_*w*_*o*_*b*_ + *p*_*e*_*o*_*e*_) + *r*_*f*_*p*_*ef*_*o*_*e*_ + *r*_*m*_(1 – *p*_*wf*_)*o*_*b*_ + *r*_*s*_*p*_*wf*_*o*_*b*_			
Focal male helps			
Direct fitness			
Focal male’s WPY	*r*_*o*_	$p_{w}=1-\frac{sg}{A}$	0
Focal male’s EPY	*r*_*o*_	$p_{e}=1-\frac{g}{A}$	0
Indirect fitness			
Social father’s EPY	*r*_*f*_	$p_{ef}=1-\frac{g}{A+d}$	*o*_*e*_
Mother’s EPY	*r*_*m*_	1 − *p*_*wf*_	*o*_*h*_
Social father and mother’s WPY	*r*_*s*_	$p_{wf}=1-\frac{sg}{A+d}$	*o*_*h*_
Inclusive fitness of helping: *r*_*f*_*p*_*ef*_*o*_*e*_ + *r*_*m*_(1 – *p*_*wf*_)*o*_*h*_ + *r*_*s*_*p*_*wf*_*o*_*h*_			

EPY, extrapair young; WPY, within-pair young.

We define a male’s probability of siring extrapair offspring as an increasing function of his age in a given breeding season, *A*. For a male that is capable of breeding for the first time as a yearling and can breed annually thereafter, *A* is also equal to age in years. It is realistic to propose that annual number of extrapair offspring sired increases with male age; for example, in western bluebirds, older males are more likely to gain EPP (Ferree and Dickinson 2014). We capture the strength of the influence of age on a male’s success at gaining paternity with the parameter *g*, which we term the “age boost.” This function can take a variety of forms and can be fitted to particular species. Here, we use $p_{e}=1-\frac{g}{A}$ , where *A* ≥ 1 and 0 < *g* ≤ 1. In this function, we capture 2 features of age-biased paternity success: 1) for a given magnitude of the age boost, the probability of siring extrapair young increases with male age and 2) for a male of a given age, the probability of siring extrapair young decreases with the magnitude of the age boost (this probability decreases much more rapidly with the age boost for younger males than for older males) (Supplementary Figure S1).

A male’s probability of siring within-pair offspring is similarly a function of his age and the magnitude of the age boost (again, a measure of how strongly age influences paternity), and this function is similarly flexible in the forms it can take, such that it can be fitted to species or populations. Here, we use $p_{w}=1-\frac{sg}{A}$ , where the parameter *s* (0 ≤ *s* ≤ 1) mediates the relationship between the probability of siring within-pair young and the probability of siring extrapair young; we call *s* the “pairing advantage.” When *s* = 1, the probabilities of siring within-pair young and extrapair young are equal; when *s* = 0, the probability of siring within-pair young is equal to 1 regardless of values of *A* and *g*. When *s* is between 0 and 1, the probability of siring within-pair young is greater than that of siring extrapair young (*p*_*w*_ > *p*_*e*_) (Supplementary Figure S2). This term, *s*, is important because it allows for tradeoffs (a negative covariance) between EPP and within-pair paternity (WPP), a positive covariance between EPP and WPP, or no relationship between EPP and WPP. This relationship can be fitted to particular species; in western bluebirds, older males do not suffer loss of paternity in their own nests as a result of gaining more EPP (Ferree and Dickinson 2014).

When a focal male is potentially helping his father and mother, the difference in age between the father and son is captured by *d*, where *d* ≥ 1 because, as is true for most birds, a father must be at least one breeding season older than his son. The father’s probability of siring extrapair young in the current breeding season is thus $p_{ef}=1-\frac{g}{A+d}$ , and his probability of siring within-pair young is $p_{wf}=1-\frac{sg}{A+d}$ .

Using these probabilities, we can build expressions for a focal male’s inclusive fitness when he breeds versus when he helps. The focal male’s direct fitness if he breeds is given by *r*_*o*_(*p*_*w*_*o*_*b*_ + *p*_*e*_*o*_*e*_), where *r*_*o*_ is the male’s relatedness to his own genetic offspring, *o*_*b*_ is the number of offspring in a brood, and *o*_*e*_ is the number of offspring a male sires if he sires extrapair offspring. Normally, *r*_*o*_ = 0.5, unless the male is inbreeding, which is rare in cooperatively breeding vertebrates (Koenig et al. 2015). If a male helps at his parents’ nest, he gains no direct fitness. Note that we assume that helper males do not gain EPP; this assumption is based on the empirical finding that identified extrapair, extragroup sires are usually breeding males, not helpers, in cooperatively breeding bird species with extrapair mating (Dickinson and Akre 1998; Richardson et al. 2001; Du and Lu 2009). Allowing helpers the possibility of gaining EPP in this model would increase the inclusive fitness benefits of helping behavior, but excluding it is more realistic, because empirically it is not a significant source of inclusive fitness.

To calculate the indirect fitness benefit of helping for the focal male, we must find the difference in offspring production in his parents’ nest if the focal male breeds versus if he helps. We assume that, if the focal male does not help, his parents will not receive help at their nest (i.e., the focal male is the only potential helper); the rarity of nests with more than one helper in the western bluebird population (2% [5/212] of nests with helpers had more than one helper) suggests that the number of potential helpers is usually limited in this system. The focal male’s helping behavior increases the success of his parents’ brood such that the number of offspring produced is *o*_*h*_, where *o*_*h*_ > *o*_*b*_. While this increase in brood productivity with help can contribute to the immediate indirect fitness benefits of helping behavior, it is not the focus of the hypothesis under study here, and empirically it is likely a minor contributor to helper inclusive fitness (Dickinson and Akre 1998; Dias et al. 2015). Regardless of whether the male breeds or helps, his father will gain a total number of extrapair offspring equal to *p*_*ef*_*o*_*e*_. If the male breeds, his mother will gain a total number of extrapair offspring equal to (1 − *p*_*wf*_)*o*_*b*_; if the male helps, his mother will instead gain (1 − *p*_*wf*_)*o*_*h*_ extrapair offspring. The offspring in the brood that are sired by the focal male’s father will number *p*_*wf*_*o*_b_ if the male breeds and *p*_*wf*_*o*_*h*_ if the male helps.

To determine the value of these offspring to the focal male, we define *r*_*s*_ as the male’s relatedness to the offspring of his social father and mother, *r*_*m*_ as the male’s relatedness to his mother’s extrapair offspring, and *r*_*f*_ as the male’s relatedness to his father’s extrapair offspring. We can incorporate the influence of the social father’s age on the probability that the social father is the focal male’s genetic father using *r*_*f*_ = 0.25 * *p*_*wd*_, where *p*_*wd*_ is the father’s probability of siring within-pair young in the year that the focal male was born (i.e., the probability that the social father is the focal male’s genetic father), which is defined by $p_{wd}=1-\frac{sg}{d}$ (note that the age difference between the father and son (*d*) is the age of the father when the son was born). The father’s probability of siring within-pair young in the year that the focal male was born is multiplied by 0.25, which is the son’s relatedness to his father’s extrapair young if his social father is also his genetic father. Thus, *r*_*f*_ captures the probability that the focal male gains indirect fitness through his social father’s reproduction. We assume that the focal male’s mother’s extrapair offspring are the male’s half-siblings, that is, that the probability that the focal male has an extrapair father that is also a close relative of his mother’s nestlings is negligible. If we also assume that a female is always the mother of all the offspring in her brood (e.g., egg-dumping does not occur), we then have the relationships *r*_*m*_ = *r*_*o*_/2 and *r*_*s*_ = *r*_*f*_ + *r*_*m*_. The first relationship comes about because the relatedness of a male to his own offspring, *r*_*o*_, represents, in the absence of inbreeding, the 50% likelihood that any randomly chosen allele in the male’s offspring is one that the male shares with his offspring. The likelihood that a randomly chosen allele in other genetic offspring of the male’s mother was inherited from the male’s mother and is shared by the male is 25%, or *r*_*o*_/2. The second relationship comes about because the likelihood that the focal male shares any randomly chosen allele in other offspring of his mother and social father is the likelihood that the allele was inherited from the focal male’s mother and is shared by the male and that offspring (*r*_*m*_) added to the likelihood that the allele was inherited from the focal male’s social father and is shared by the focal male and that offspring (*r*_*f*_). We employ the relationships *r*_*m*_ = *r*_*o*_/2 and *r*_*s*_ = *r*_*f*_ + *r*_*m*_ in all analyses.

The fitness the focal male gains through siblings and half-siblings if he breeds is *r*_*s*_*p*_*wf*_*o*_*b*_ + *r*_*m*_(1 – *p*_*wf*_)*o*_*b*_ + *r*_*f*_*p*_*ef*_*o*_*e*_, and the fitness he gains through siblings and half-siblings if he helps is *r*_*s*_*p*_*wf*_*o*_*h*_ + *r*_*m*_ (1 – *p*_*wf*_)*o*_*h*_ + *r*_*f*_*p*_*ef*_*o*_*e*_. Finding the difference between these 2 quantities gives the indirect fitness benefit of helping behavior, (*o*_*h*_ − *o*_*b*_) ((1 – *p*_*wf*_)*r*_*m*_ + *p*_*wf*_*r*_*s*_).

The male’s inclusive fitness when he breeds at age *A*, *w*_*bA*_, is equal to his direct fitness, as he does not gain an indirect fitness benefit without helping; his inclusive fitness when he helps at age *A*, *w*_*hA*_, is equal to the indirect fitness benefit of helping, as he gains no direct fitness:

(1a) $$w_{bA}=r_{o}\left(p_{w}o_{b}+p_{e}o_{e}\right)$$

(1b) $$w_{hA}=\left(o_{h}-o_{b}\right)\left(\left(1-p_{wf}\right)r_{m}+p_{wf}r_{s}\right)$$

### Age-biased paternity model with load-lightening

Food delivery by helpers can result in either 1) additive provisioning, wherein the breeding male and female feed at the same rate with or without a helper or 2) compensatory provisioning, wherein the male and female reduce their feeding rates in the presence of a helper (Dickinson et al. 1996; Hatchwell and Russell 1996; Kingma et al. 2010). Reductions in breeder provisioning rates can be associated with increased survivorship (Kingma et al. 2010). Even when provisioning is compensatory, the sum of all feeding trips may exceed the number of trips made by a pair breeding without a helper (Dickinson et al. 1996; Hatchwell and Russell 1996; Kingma et al. 2010). Thus, load-lightening and current indirect fitness benefits of helping through increased provisioning may co-occur (Dickinson et al. 1996; Kingma et al. 2010). In this model, we separate the effects of load-lightening and increased provisioning: the load-lightening parameter captures the effect of helping on annual mortality, and the effects of increased food delivery are captured in the difference between *o*_*h*_, the number of offspring produced with help, and *o*_*b*_, the number of offspring produced in a brood without help.

We add the load-lightening effect of help to the basic model as follows. Each adult that breeds in the absence of a helper suffers annual mortality with probability *m*_*b*_. Each adult that raises offspring in a group that includes a helper, whether the adult is a breeder or a helper, suffers annual mortality with probability *m*_*h*_, where *m*_*h*_ = *L* * *m*_*b*_ and *L* is a parameter capturing the effect of load-lightening on mortality. When *L* = 1, adults raising offspring with help suffer mortality equal to adults raising offspring without help, that is, load-lightening does not occur or does not influence survival. When *L* < 1, adults raising offspring with help have a lower mortality probability than adults raising offspring without help, meaning that load-lightening positively influences survival. When a male breeds, both he and his parents suffer the mortality probability *m*_*b*_. When a male helps, both he and parents suffer the mortality probability *m*_*h*_. This is reasonable for western bluebirds, because helpers share equally in feeding young (Dickinson et al. 1996).

We do not include reproductive senescence in this model: we assume that individuals are able to continue producing offspring until they die. In western bluebirds, older males are more likely to gain EPP, and successful extrapair males are no less likely to sire within-pair young (Ferree and Dickinson 2011, 2014). We also assume that mortality probability does not increase with age (survival senescence). Based on previous cross-species analyses, some bird species may have low rates of survival senescence (Jones et al. 2008). To capture those systems, this assumption could be relaxed by making the mortality probability (*m*_*b*_) a function of age. In western bluebirds, which live for up to 9 years, there is no evidence of senescence in either survival or annual reproduction (Dickinson JL, Stern CA, unpublished analyses).

Here, we focus on identifying the conditions under which a male that helps in his first year as an adult has higher lifetime fitness than a male that breeds in his first year as an adult, given that both males subsequently breed in every remaining breeding season in their lifetimes. This approach allows us to estimate the lifetime fitness effects of delaying the onset of breeding in favor of helping. The male that helps gain fitness *w*_*h*1_ in his first year, and the male that breeds gains fitness *w*_*b*1_ in his first year (age *A* = 1; Equations 1a and 1b). In the subsequent years, each male gains fitness according to his age: *w*_*b*2_ in his second year as an adult, *wb*_3_ in his third year as an adult, and so on. However, these subsequent fitness gains are modified by the probability of survival, as follows. A male that helped in his first year has probability 1 − *m*_*h*_ of surviving to his second year. He breeds in his second year, and thus has probability 1 − *m*_*b*_ of surviving from his second to his third year; however, he can only reach his third year if he has survived to his second year, so his total probability of gaining the benefit of breeding in his third year is (1 − *m*_*h*_) (1 − *m*_*b*_). Thus, his lifetime fitness if he dies after his third year as an adult is *w*_*h*1_ + (1 – *m*_*h*_)*w*_*b*2_ + (1 – *m*_*h*_) (1 – *m*_*b*_)*w*_*b*3_. By the same logic, the lifetime fitness of a male who bred in his first year and died after his third year as an adult is *w*_*b*1_ + (1 – *m*_*b*_)*w*_*b*2_ + (1 – *m*_*b*_) (1 – *m*_*b*_)*w*_*b*3_. More generally, we can write the lifetime fitness of a male who survives to age *A* after helping in his first year as

(2a) $$W_{hA}=w_{h1}+\sum_{A=2}\left(1-m_{h}\right){\left(1-m_{b}\right)}^{j}w_{bA}$$

where *j* = *A* − 2. Similarly, we can write the lifetime fitness of a male who survives to age *A* after breeding in his first year as

(2b) $$W_{bA}={\sum_{A=1}\left(1-m_{b}\right)}^{k}w_{bA}$$

where *k* = *A* − 1.

### Parameterizing the model with western bluebird data

The fitness functions can be parameterized with data from specific study systems; here, we parameterize the model using data from a long-term study of western bluebirds in central-coastal California (Dickinson et al. 1996). In the western bluebird population, nests with adult helpers fledge on average 3.56 offspring, while nests without helpers fledge on average 2.89 offspring (Dickinson and Akre 1998). Those males that sire extrapair offspring sire on average 1.87 extrapair young (Ferree and Dickinson 2014). The relatedness between a breeding female and her offspring is 0.49 on average, supporting the assumption that a female is the mother of all offspring in her brood (Dickinson and Akre 1998). Western bluebirds suffer approximately 50% annual mortality (Dickinson et al. 1996). We thus parameterize the model as follows: *o*_*b*_ = 2.89, *o*_*h*_ = 3.56, *o*_*e*_ = 1.87, *r*_*o*_ = 0.5, *m*_*b*_ = 0.5.

### Analyses

First, we seek to identify the conditions under which helping is favored over breeding, when the age-biased paternity model without load-lightening is parameterized with western bluebird data. Using Equations 1a and 1b, we search for the region in which a male that helps in his first year as an adult gains greater inclusive fitness than a male that breeds in his first year as an adult (i.e., *w*_*h*1_ > *w*_*b*1_ ). This region is specified by parameters *g*, *s*, and *d*. Next, we study the age-biased paternity model with load-lightening, evaluating Equations 2a and 2b at a range of possible lifespans to determine how male lifespan and reduced annual mortality for adults in a group with a helper influence the range of conditions under which helping in the first year is favored over breeding in the first year (i.e., *W*_*hA*_ > *W*_*bA*_).

## RESULTS

### Age-biased paternity model without load-lightening

**Figure 1:**
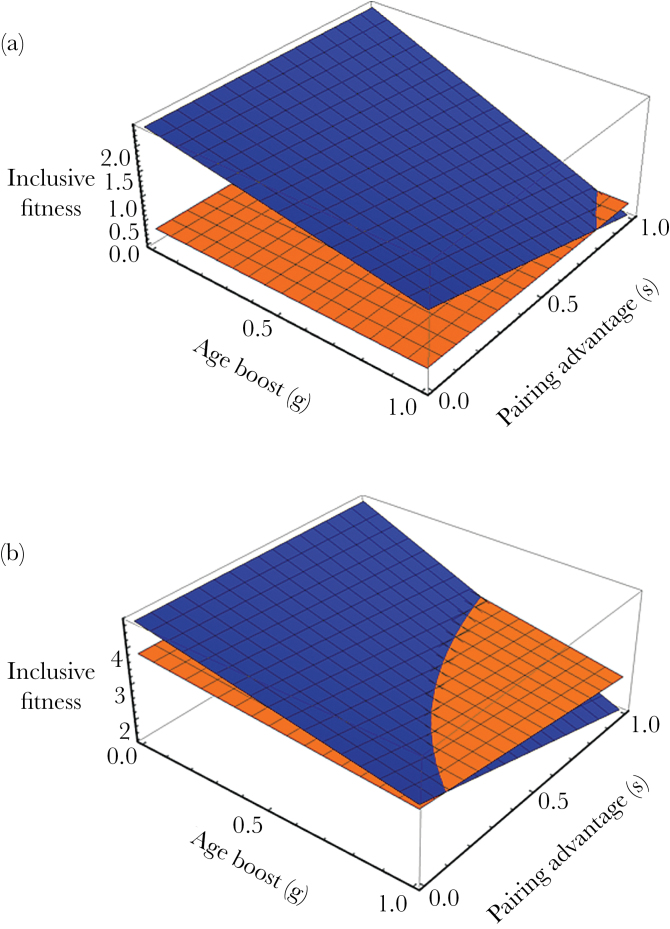
The inclusive fitness of a focal male that either breeds independently (dark blue surface) or helps at his parents’ nest (light orange surface) in his first year as an adult, (a) in the age-biased paternity model with *d* = 3, or (b) over the male’s 8-year lifespan in the age-biased paternity model with load-lightening (*d* = 3 and *L* = 0.5; assuming that the male breeds in every year after his first year as an adult). The model is parameterized using data from western bluebirds: *o*_*b*_ = 2.89, *o*_*h*_ = 3.56, *o*_*e*_ = 1.87, *r*_*o*_ = 0.5, and *m*_*b*_ = 0.5 (Dickinson et al. 1996; Dickinson and Akre 1998; Ferree and Dickinson 2014).

We find that helping yields greater fitness benefits than breeding in a small region of parameter space, when older males are much more likely to gain EPP than younger males (*g* is high) and a male is not much more likely to sire within-pair young than he is to sire extrapair young (*s* is high). Figure 1a shows the inclusive fitness surfaces of helping and breeding when the age difference between father and son is 3 years. The influence of age on paternity success is known to be strong in western bluebirds (Ferree and Dickinson 2011, 2014). Males are more likely to sire within-pair than extrapair young in western bluebirds: about 20% of males sire extrapair young while about 50% lose WPP to another male (Ferree and Dickinson 2014). Thus, we expect that the region of parameter space in which *g* is high is realistic, but *s* is likely moderate rather than high in western bluebirds.

### Age-biased paternity model with load-lightening

Adding load-lightening to the age-biased paternity model allows us to identify the conditions under which a male that helps in his first year as an adult has higher lifetime fitness than a male that breeds in his first year as an adult, given that both males subsequently breed in every remaining breeding season in their lifetimes. The size of the region in which helping is favored over breeding in the first year as an adult increases with male lifespan. Figure 1b shows this region for a male lifespan of 7 adult breeding seasons (8 years of life); the set of high *g* and moderate *s* values for which helping is favored over breeding is likely realistic for western bluebirds.

**Figure 2:**
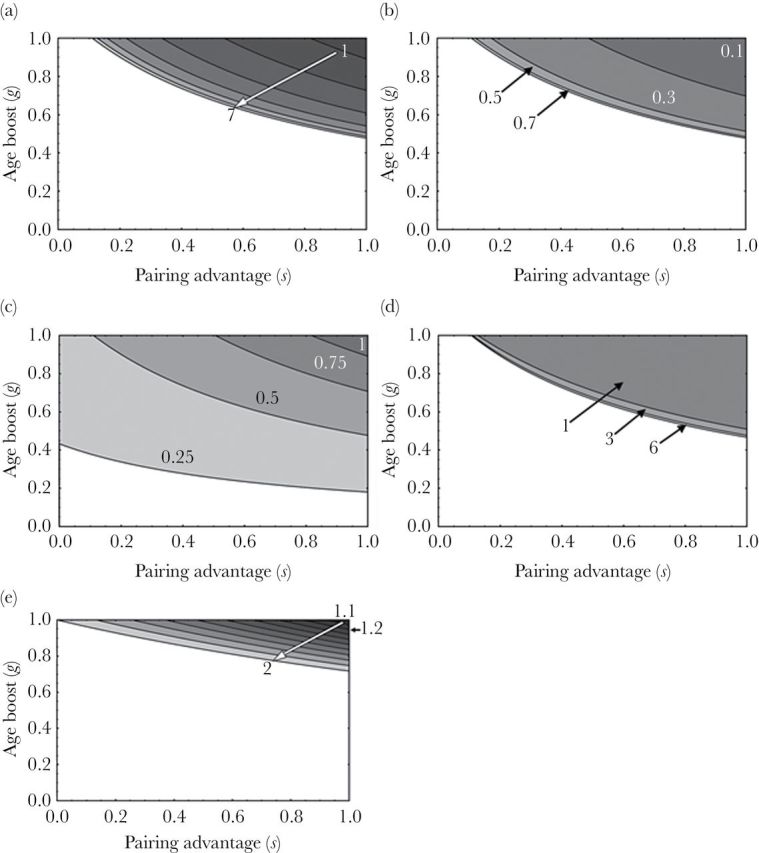
The effect on the size of the region (shaded) in which helping in the first year as an adult yields greater fitness than breeding in the first year as an adult of: (a) the focal male’s lifespan, when the focal male breeds in every subsequent year as an adult, over male lifespans from 2 (1 breeding season as an adult) to 8 (7 breeding seasons as an adult) years; (b) annual mortality probability (*m*_*b*_); (c) the proportion of the annual mortality probability suffered by adults in a group with a helper (*L*); (d) the age difference between the father and the focal male (*d*); and (e) the ratio of offspring number produced by a group with a helper to offspring number produced by a pair without a helper (*o*_*h*_/*o*_*b*_; the arrow at the ratio 1.2 indicates the approximate position of the western bluebird system, in which *o*_*h*_/*o*_*b*_ = 1.23). The model is parameterized using data from western bluebirds for *r*_*o*_, *m*_*b*_, *o*_*e*_, *o*_*b*_, and *o*_*h*_ (Dickinson et al. 1996; Dickinson and Akre 1998; Ferree and Dickinson 2014). In all panels, *o*_*e*_ = 1.87 and *r*_*o*_ = 0.5. In panels (b), (c), (d), and (e), male lifespan is 8 years (7 breeding seasons); we varied male lifespan in panel (a). In panels (a), (c), (d), and (e), *m*_*b*_ = 0.5; we varied *m*_*b*_ in panel (b). In panels (a), (b), (d), and (e), *L* = 0.5; we varied *L* in panel (c). In panels (a), (b), (c), and (e), *d* = 3; we varied *d* in panel (d). In panels (a), (b), (c), and (d), *o*_*b*_ = 2.89 and *o*_*h*_ = 3.56; we varied the ratio *o*_*h*_/*o*_*b*_ in panel (e).

Varying the model’s parameters one by one reveals their effects on the likelihood that helping is favored over breeding. As shown in Figure 2, we find that the size of the region in which helping in the first year as an adult yields greater fitness than breeding in the first year as an adult 1) increases with focal male lifespan, 2) increases with annual mortality probability, 3) decreases as the proportion of annual mortality suffered by adults in a group with a helper increases, 4) increases with the age difference between father and son, and 5) increases as the ratio of offspring number produced by a group with a helper to offspring number produced by a pair increases. Thus, helping is more likely to be favored over breeding when males have longer life expectancies, adults have a higher likelihood of dying each year, adults in a group with a helper have lower annual mortality probability than adults that breed without a helper, the age difference between father and son is large, and groups with a helper produce substantially more offspring than do pairs without a helper. However, we note that some of these effects are relatively small; for example, the age difference between father and son has only a minor influence on the size of the region in which helping is favored over breeding, and the effects of male lifespan and annual mortality probability attenuate as they increase.

## DISCUSSION

The current function of helping behavior in bird species with high rates of EPP is difficult to explain by invoking the indirect benefits helpers gain through caring for non-descendent kin alone. Here, we use a proof-of-concept model to test the idea that the combination of age-biased paternity success, wherein older males are more successful extrapair sires, and load-lightening, wherein adults in groups with a helper have reduced annual mortality, can lead to fitness benefits of helping that outweigh the fitness gains from independent breeding. We analyze the model with parameters based on data from a population of western bluebirds in which EPP success is age-biased, 7% (3–16%) of pairs have a helper, and males successful at EPP have double the reproductive success of males that are not (Dickinson et al. 1996; Dickinson and Akre 1998; Ferree and Dickinson 2014). Our results demonstrate that an age bias in paternity success can lead to greater inclusive fitness from helping than from breeding for first-year adult males, and that a reduction in annual adult mortality for adults in groups with a helper adds to this effect, increasing the area of parameter space in which helping is favored over breeding. These results suggest that long-term studies are vital to understanding cooperative breeding systems and can be greatly enhanced with expansion of parentage data to allow for analysis of the lifetime fitness effects of helping in species with EPP.

Comparing the inclusive fitness benefits of helping versus breeding when age-biased paternity, but not load-lightening, is included in the model, we find that helping is favored over breeding when age strongly influences paternity success (*g* is high) and a male is not much more likely to sire within-pair young than he is to sire extrapair young (*s* is high). When a breeding male does not have a strong WPP advantage, this means that more of his paternity is subject to regulation by the age boost, explaining why high *s* results in a larger advantage to helping when the age boost is also strong. The region of parameter space in which both of these effects are sufficiently strong to elevate the inclusive fitness of helping over that of breeding is relatively small, and the high required *s* value is likely not realistic for western bluebirds (although the high *g* likely is realistic; Ferree and Dickinson 2011, 2014). However, this result is still important, because it demonstrates that load-lightening is not required to favor helping over breeding in some cases.

Adding load-lightening to the model and evaluating male fitness over a multiyear lifespan increases the size of the region in which the inclusive fitness benefits of helping in the first year as an adult outweigh those of breeding. The more load-lightening reduces annual mortality, and the longer males can expect to live, the larger are the inclusive fitness benefits of helping in the first year as an adult. Even moderate values of the age boost (*g*) and pairing advantage (*s*), including values that are likely realistic for western bluebirds, are sufficient to favor helping over breeding as long as load-lightening has a substantial effect on annual mortality and males can expect to live for multiple seasons as adults. The association between longevity and cooperative breeding in birds has been recognized for some time (Russell 1989; Arnold and Owens 1998); our result that longer lifespans allow for the accumulation of the benefits of helping over time is consistent with this pattern. Our findings provide an explanation for the positive relationship between promiscuity and lifespan in cooperatively breeding birds (Downing et al. 2015) and emphasize the importance of studying helping behavior in a life-history context (Rodrigues and Kokko 2016).

We find that the age difference between father and son influences whether helping confers higher inclusive fitness benefits than breeding, but only to a minor extent. Based on the model, we therefore expect that population-level characteristics including male lifespan, the effect of age on EPP success, and the effect of helping on annual mortality will more strongly influence helping behavior than the age difference between father and son. Considering the western bluebirds in particular, we suggest that the parameter ranges in which helping is favored over breeding in the model are likely realistic for this system; however, more data on the lifetime inclusive fitness of males, including formal survival analysis and EPP success, would illuminate the extent to which delayed extrapair benefits are important for helping in this species.

We have focused in this paper on illustrating the logic of the delayed extrapair benefits hypothesis and have not taken into account additional factors such as the age structure of the population. However, the current model could be expanded to the population level, which would allow for consistency in, for example, the equivalence of paternity number and maternity number, ensuring that the model takes into account that every offspring has only one mother and one father (Houston and McNamara 2002; Kokko 2003). While we know that the age structure of the western bluebird population allows males to enjoy increased success in gaining EPP as they age (Ferree and Dickinson 2011, 2014), exploring mathematically how the age structure of a population influences the extent to which older males can actually achieve higher EPP success is an exciting avenue for future research. Understanding the influence of the adult sex ratio, which is male-biased in western bluebirds (Dickinson 2004b), on the importance of age-biased paternity, is another important future direction.

In summary, the model predicts that helping will occur most frequently in populations with high levels of EPP when 1) older males are much more likely to gain EPP than younger males, 2) a male is not much more likely to sire within-pair young than he is to sire extrapair young, 3) males live for multiple breeding seasons, and 4) helping substantially reduces annual mortality for helpers and their parents. Determining the extent to which populations of cooperatively breeding birds with EPP match these characteristics is an important direction for future research and will reveal the scope of the logical framework tested in this model.

Although previous studies have shown that monogamy was important in the evolution of cooperative breeding (Hughes et al. 2008; Cornwallis et al. 2010; Lukas and Clutton-Brock 2012), explaining the current function of helping behavior in populations with high levels of EPP is an outstanding challenge. Here, we have presented a hypothesis to explain this phenomenon and demonstrated mathematically that the logic of the delayed extrapair benefits hypothesis holds: age-dependent success in EPP can enhance the inclusive fitness advantages of helping behavior. Both testing the general predictions of the model and estimating its parameters require long-term data from cooperatively breeding bird populations in which genetic paternity and lifetime reproductive success are known, emphasizing the importance of detailed, long-term studies in understanding cooperative breeding systems.

## SUPPLEMENTARY MATERIAL

Supplementary material can be found at Supplementary Data

## FUNDING

C.A.S. is supported by an Omidyar Fellowship at the Santa Fe Institute. The western bluebird project was supported by the Cornell Lab of Ornithology and by the National Science Foundation (grant numbers IOS 0097027 and IOS 0718416 to J.L.D.).

## Supplementary Material

Supplementary Data

Supplementary Data
