# Supplementary material for: Effects of load-lightening and delayed extrapair benefits on the fitness consequences of helping behavior
Source: Behav Ecol. 2016 Feb 17;27(4):1078–86. doi: 10.1093/beheco/arw018 (PMC6191074; doi:10.1093/beheco/arw018)
Supplement: Supplementary Data [file arw018_supplementary_data.zip › Supplementary_Material_Stern_and_Dickinson_PDF.pdf]

Effects of load-lightening and delayed extra-pair benefits on the fitness consequences of helping behavior

Caitlin A. Stern and Janis L. Dickinson

Supplementary material: specification and analysis of models, generation of main text figures 1 and 2, and supplementary figures S1 and S2

Age-biased paternity

A = age of focal male in a given breeding season (A >= 1)

g = age effect (0 < g <= 1)

s = pairing advantage (0 <= s <= 1)

p_e = focal male's probability of siring extra-pair offspring = 1 - g/A

p_w = focal male's probability of siring within-pair offspring = 1- (s*g)/A

d = age difference between father and son

p_{ef} = father's probability of siring extra-pair young = 1 - g/(A+d)

p_{wf} = father's probability of siring within-pair young = 1 - (s*g)/(A+d)

p_{wd} = father's probability of siring within-pair young the year the focal male was born = 1 - (s*g)/(d)

r_o = focal male's relatedness to his own genetic offspring

o_b = number of offspring in a brood without a helper

o_e = number of offspring a male sires if he sires extra-pair offspring

r_f = relatedness of focal male to father's extra-pair offspring; 0.25*p_{wd}

r_m = relatedness of focal male to mother's extra-pair offspring; r_o/2

Focal male's inclusive fitness from breeding at age A:

```
IFbA = FullSimplify[ro * (pw * ob + pe * oe) + rf * pef * oe + rm * (1 - pwf) * ob + rs * pwf * ob]
```

$$\frac{1}{A\,(A+d)}\left(o_e\,(A\,(A+d-g)\,r_f+(A+d)\,(A-g)\,r_o)+o_b\,(A\,g\,s\,r_m+(A+d)\,(A-g\,s)\,r_o+A\,(A+d-g\,s)\,r_s)\right)$$

Focal male’s inclusive fitness from helping at age A:

```
IFhA = FullSimplify[rf * pef * oe + rm * (1 - pwf) * oh + rs * pwf * oh]
```

$$\frac{1}{A+d}\left((A+d-g)\,o_e\,r_f+o_h\,(g\,s\,r_m+(A+d-g\,s)\,r_s)\right)$$

Indirect fitness benefit of helping behavior:

```
FullSimplify[
 (rf * pef * oe + rm * (1 - pwf) * oh + rs * pwf * oh) - (rf * pef * oe + rm * (1 - pwf) * ob + rs * pwf * ob)]
```

$$-\frac{(o_b-o_h)\,(g\,s\,r_m+(A+d-g\,s)\,r_s)}{A+d}$$

Write this using $(o_h - o_b)$ as the first term to remind the reader that this is the benefit of helping behavior. Check that the expressions for the indirect fitness benefit of helping are equivalent:

```
Reduce[(ob - oh) ((-1 + pwf) rm - pwf rs) == (oh - ob) * ((1 - pwf) rm + pwf rs)]
```

True

Focal male’s fitness gain from breeding at age A is equal to his direct fitness:

```
wbA = FullSimplify[ro * (pw * ob + pe * oe)]
```

$$\frac{((A-g\,s)\,o_b+(A-g)\,o_e)\,r_o}{A}$$

Focal male’s fitness gain from helping at age A is equal to the indirect fitness benefit of helping:

```
whA = FullSimplify[(oh - ob) * ((1 - pwf) rm + pwf rs)]
```

$$-\frac{(o_b-o_h)\,(g\,s\,r_m+(A+d-g\,s)\,r_s)}{A+d}$$

Employ simplifying assumptions:

```
whA = FullSimplify[whA /. rs → rm + rf /. rf → 0.25 * pwd /. rm → (ro / 2)]
wbA = FullSimplify[wbA /. rs → rm + rf /. rf → 0.25 * pwd /. rm → (ro / 2)]
```

$$-\frac{1}{A+d}\left(o_b - o_h\right)\left(\frac{1}{2} g\, s\, r_o + \frac{1}{d} 0.5\,(A + d - 1.\, g\, s)\,(0.5\, d - 0.5\, g\, s + d\, r_o)\right)$$

$$\frac{((A - g\, s)\, o_b + (A - g)\, o_e)\, r_o}{A}$$

Define paternity probabilities:

```
pe = 1 - (g / A);
pw = 1 - ((s * g) / A);
pef = 1 - g / (A + d);
pwf = 1 - (s * g) / (A + d);
pwd = 1 - ((s * g) / d);
```

Figure S1: the influence of age boost (*g*) magnitude on the probability of gaining extra-pair paternity (p_e)

As the magnitude of the age boost decreases, the probability that a first-year male (A=1) gains extra-pair paternity (EPP) increases, and the difference between the probability of gaining EPP at A=1 and A=7 decreases.

```
g025pe = pe /. g → 0.25; g05pe = pe /. g → 0.5; g075pe = pe /. g → 0.75; g1pe = pe /. g → 1;
```

```
Plot[{g025pₑ, g05pₑ, g075pₑ, g1pₑ}, {A, 1, 7}, PlotStyle →
  {{Thickness[0.006], GrayLevel[0.7]}, {Thickness[0.006], GrayLevel[0.5]},
   {Thickness[0.006], GrayLevel[0.3]}, {Thickness[0.006], Black}},
 PlotLegend → {"pₑ with g=0.25", "pₑ with g=0.5", "pₑ with g=0.75", "pₑ with g=1"},
 LegendPosition → {0.3, -0.3}, LegendShadow → None,
 LegendSize → {0.5, 0.5}, LabelStyle → Directive[Black, FontSize → 22],
 ImageSize → Large, AxesStyle → Directive[Thick, Black],
 PlotRange → {{1, 7}, {0.0, 1.0}}, Frame → {{True, False}, {True, False}},
 FrameLabel → {"Age in adult breeding seasons (A)", "Prob. of gaining EPP (pₑ)"}]
```

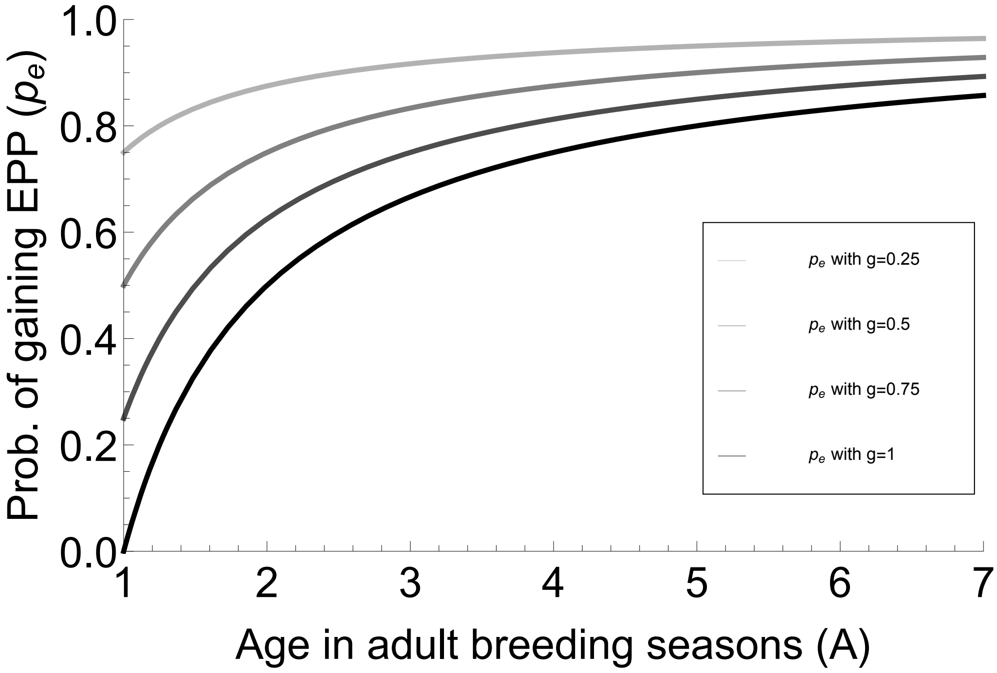

Figure S2: the relationship between the probability of gaining extra-pair young (p_e) and that of gaining within-pair young (p_w) is mediated by the pairing advantage (s)

When s=1, the probability of gaining extra-pair young (p_e; gray line) and the probability of gaining within-pair young (p_w; dotted line) are equal; when s < 1, the probability of gaining extra-pair young (p_e) is lower than that of gaining within-pair young (p_w; black line and dashed lines).

```
g05s025pw = pw /. g → 0.5 /. s → 0.25; g05s05pw = pw /. g → 0.5 /. s → 0.5;
g05s075pw = pw /. g → 0.5 /. s → 0.75; g05s1pw = pw /. g → 0.5 /. s → 1;
```

```
Plot[{g05pₑ, g05s025p_w, g05s05p_w, g05s075p_w, g05s1p_w}, {A, 1, 7},
 PlotStyle → {{Thickness[0.006], GrayLevel[0.5]}, {Thickness[0.006], Black},
   {Thickness[0.006], Black, Dashing[Large]}, {Thickness[0.006], Black,
    Dashing[Medium]}, {Thickness[0.006], Black, Dashing[Small]}},
 LabelStyle → Directive[Black, FontSize → 22], ImageSize → Large,
 AxesStyle → Directive[Thick, Black], PlotRange → {{1, 7}, {0.0, 1.0}},
 PlotLegend → {"pₑ g=0.5", "p_w g=0.5, s=0.25",
   "p_w g=0.5, s=0.5", "p_w g=0.5, s=0.75", "p_w g=0.5, s=1"},
 LegendPosition → {0.3, -0.3}, LegendShadow → None, LegendSize → {0.5, 0.5},
 Frame → {{True, False}, {True, False}}, FrameLabel →
  {"Age in adult breeding seasons (A)", "Paternity probability (pₑ or p_w)"}]
```

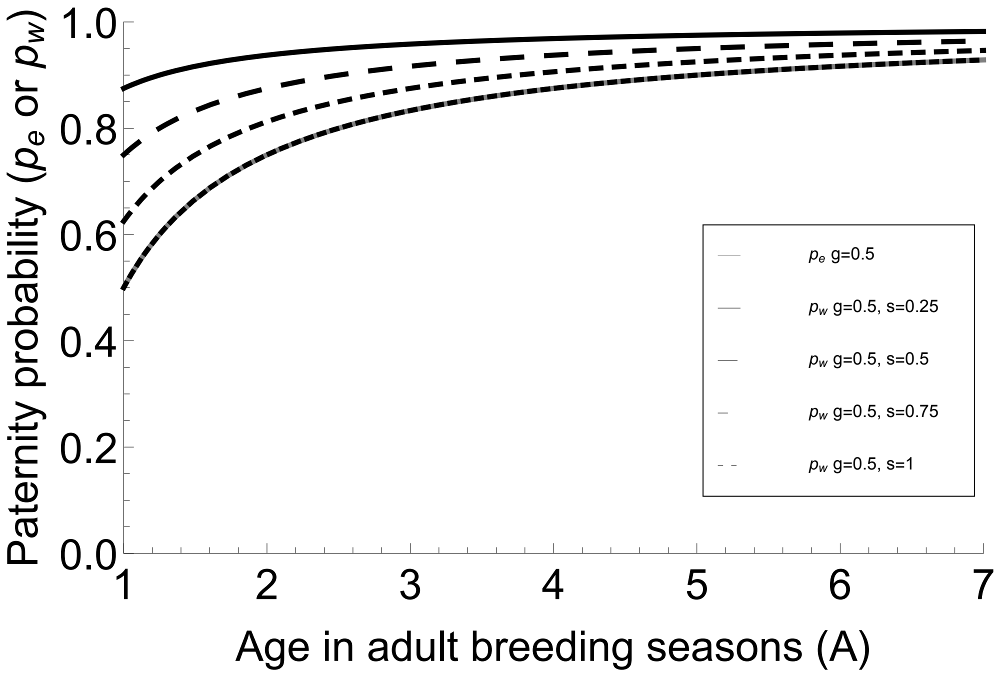

Figure 1a)

Focal male's fitness gain from breeding and helping at age 1:

```
w_b1 = FullSimplify[wbA /. A → 1]
w_h1 = FullSimplify[whA /. A → 1]
```

$$((1 - g\, s)\, o_b - (-1 + g)\, o_e)\, r_o$$

$$-\frac{1}{1+d}(o_b - o_h)\left(\frac{1}{2} g\, s\, r_o + \frac{1}{d} 0.5\,(1 + d - 1.\, g\, s)\,(0.5\, d - 0.5\, g\, s + d\, r_o)\right)$$

Focal male's fitness from breeding at age 1, parameterized with western bluebird data:

```
paramw_b1 = FullSimplify[w_b1 /. r_o → 0.5 /. o_b → 2.89 /. o_h → 3.56 /. o_e → 1.87]
```

$2.38 + g\,(-0.935 - 1.445\,s)$

Focal male's fitness from helping at age 1, parameterized with western bluebird data:

```
paramw_h1 = FullSimplify[w_h1 /. r_o → 0.5 /. o_b → 2.89 /. o_h → 3.56 /. o_e → 1.87]
```

$$\frac{1}{1+d}\,0.67\left(0.5 + 0.5\,d - 0.5\,g\,s + \frac{g\,s\,(-0.25 + 0.25\,g\,s)}{d}\right)$$

Under what conditions do the fitness benefits of helping in the first year as an adult exceed those of breeding in the first year as an adult?

```
Plot3D[{paramw_b1 /. d → 3 /. h_1 → 0, paramw_h1 /. d → 3 /. h_1 → 1}, {g, 0, 1}, {s, 0, 1},
  PlotStyle → {Blue, Orange}, LabelStyle → Directive[Black, FontSize → 22],
  ImageSize → Large, AxesStyle → Directive[Thick, Black], ViewPoint -> {2, -2.5, 2}]
```

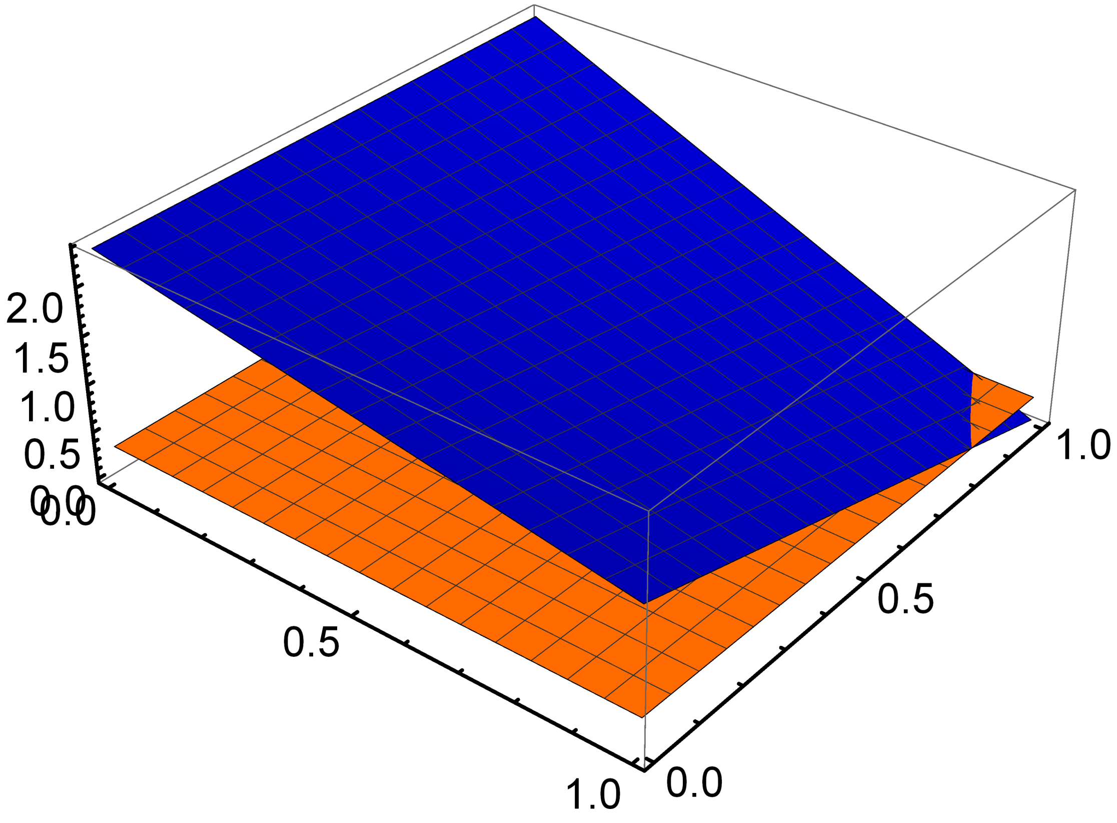

Adding load-lightening to the age-biased paternity model

m_b = mortaility rate of a breeder without a helper

m_h = mortaility rate of breeders and helper in a group with a helper = m_b*L

```
mh = mb * L
```

$L\,mb$

Calculate the lifetime fitness of a male that helps in his first year and breeds in every subsequent year, living to age 7:

```
j = A - 2;
wLh7 = Simplify[wh1 + Sum[(1 - mh) * ((1 - mb) ^j) * wbA, {A, 2, 7}]]
```

$$-\frac{1}{7}(-1+mb)^5(-1+L\,mb)((-7+g\,s)\,o_b+(-7+g)\,o_e)\,r_o+\frac{1}{6}(-1+mb)^4(-1+L\,mb)((-6+g\,s)\,o_b+(-6+g)\,o_e)\,r_o-\frac{1}{5}(-1+mb)^3(-1+L\,mb)((-5+g\,s)\,o_b+(-5+g)\,o_e)\,r_o+\frac{1}{4}(-1+mb)^2(-1+L\,mb)((-4+g\,s)\,o_b+(-4+g)\,o_e)\,r_o-\frac{1}{3}(-1+mb)(-1+L\,mb)((-3+g\,s)\,o_b+(-3+g)\,o_e)\,r_o+\frac{1}{2}(-1+L\,mb)((-2+g\,s)\,o_b+(-2+g)\,o_e)\,r_o-\frac{1}{1+d}(o_b-o_h)\left(\frac{1}{2}g\,s\,r_o+\frac{1}{d}0.5\,(1+d-1.\,g\,s)\,(0.5\,d-0.5\,g\,s+d\,r_o)\right)$$

Calculate the lifetime fitness of a male that breeds in his first year and in every subsequent year, living to age 7:

```
k = A - 1;
wLb7 = Sum[((1 - mb) ^k) * wbA, {A, 1, 7}]
```

$$((1-g\,s)\,o_b+(1-g)\,o_e)\,r_o+\frac{1}{2}(1-mb)((2-g\,s)\,o_b+(2-g)\,o_e)\,r_o+\frac{1}{3}(1-mb)^2((3-g\,s)\,o_b+(3-g)\,o_e)\,r_o+\frac{1}{4}(1-mb)^3((4-g\,s)\,o_b+(4-g)\,o_e)\,r_o+\frac{1}{5}(1-mb)^4((5-g\,s)\,o_b+(5-g)\,o_e)\,r_o+\frac{1}{6}(1-mb)^5((6-g\,s)\,o_b+(6-g)\,o_e)\,r_o+\frac{1}{7}(1-mb)^6((7-g\,s)\,o_b+(7-g)\,o_e)\,r_o$$

Parameterize with western bluebird data:

```
wLh7WEBL = Simplify[wLh7 /. rₒ → 0.5 /. ob → 2.89 /. oh → 3.56 /. oe → 1.87 /. mb → 0.5]
wLb7WEBL = Simplify[wLb7 /. rₒ → 0.5 /. ob → 2.89 /. oh → 3.56 /. oe → 1.87 /. mb → 0.5]
```

$$\frac{1}{d\,(1.+d)}\Big(g\,s\,(-0.1675+0.1675\,g\,s)+ d\,(5.02063-2.34281\,L+g\,(-0.71906+0.35953\,L-1.44627\,s+0.555637\,L\,s))+ d^2\,(5.02063-2.34281\,L+g\,(-0.71906+0.35953\,L-1.11127\,s+0.555637\,L\,s))\Big)$$

$$4.72281+g\,(-1.29453-2.00064\,s)$$

Figure 1b)

```
Plot3D[{wLh7WEBL /. d → 3 /. L → 0.5, wLb7WEBL /. d → 3 /. L → 0.5}, {g, 0, 1}, {s, 0, 1},
 PlotStyle → {Orange, Blue}, LabelStyle → Directive[Black, FontSize → 22],
 ImageSize → Large, AxesStyle → Directive[Thick, Black], ViewPoint -> {2, -2.5, 2}]
```

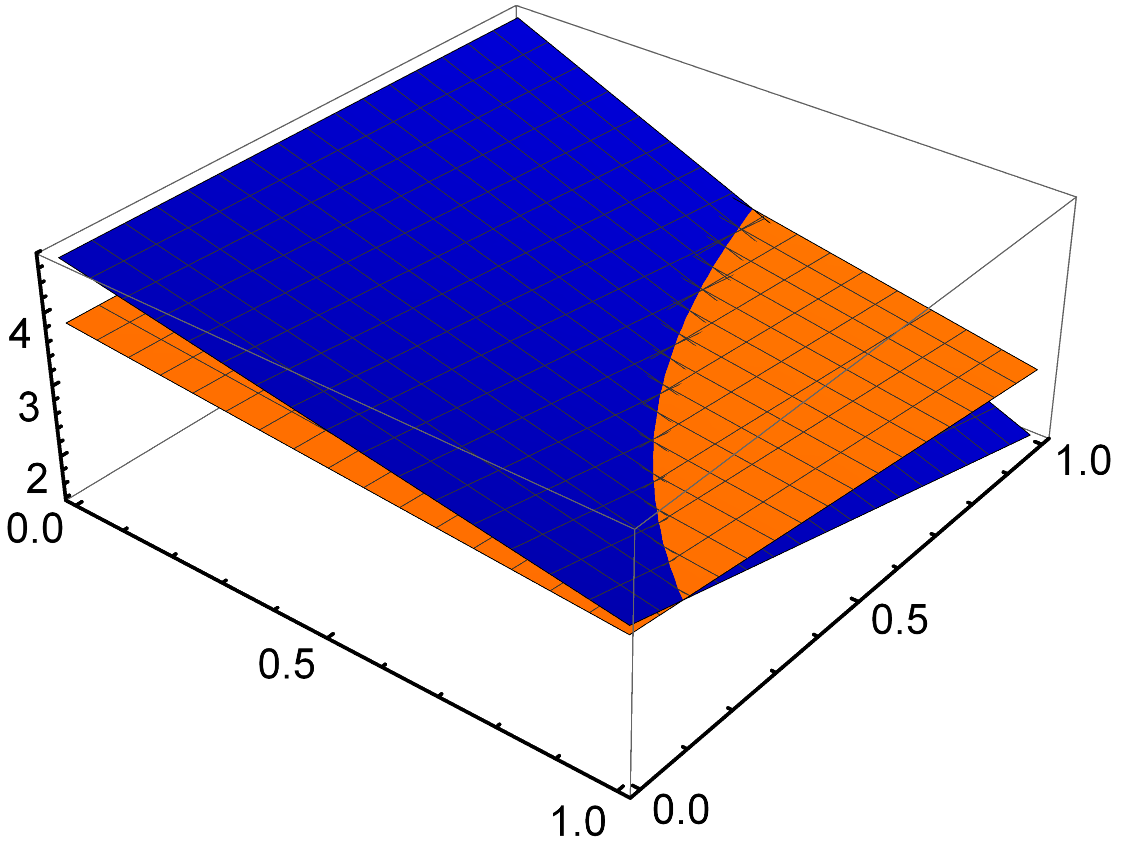

Check that this formulation re-captures the results for the first breeding season only:

```
j = A - 2;
```

```
wLh1 = Simplify[wh1]
```

$$-\frac{1}{1+d}(o_b - o_h)\left(\frac{1}{2}\, g\, s\, r_o + \frac{1}{d}\, 0.5\,(1 + d - 1.\, g\, s)\,(0.5\, d - 0.5\, g\, s + d\, r_o)\right)$$

```
k = A - 1;
```

```
wLb1 = Sum[((1 - mb)^k) * wbA, {A, 1, 1}]
```

$$((1 - g\, s)\, o_b + (1 - g)\, o_e)\, r_o$$

```
wLh1WEBL = Simplify[wLh1 /. ro → 0.5 /. ob → 2.89 /. oh → 3.56 /. oe → 1.87 /. mb → 0.5]
wLb1WEBL = Simplify[wLb1 /. ro → 0.5 /. ob → 2.89 /. oh → 3.56 /. oe → 1.87 /. mb → 0.5]
```

$$\frac{1}{1+d}\, 0.67\left(0.5 + 0.5\, d - 0.5\, g\, s + \frac{g\, s\,(-0.25 + 0.25\, g\, s)}{d}\right)$$

$$2.38 + g\,(-0.935 - 1.445\, s)$$

```
Plot3D[{wLh1WEBL /. d → 3 /. L → 0.5, wLb1WEBL /. d → 3 /. L → 0.5}, {g, 0, 1}, {s, 0, 1},
 PlotStyle → {Orange, Blue}, LabelStyle → Directive[Black, FontSize → 22],
 ImageSize → Large, AxesStyle → Directive[Thick, Black], ViewPoint -> {2, -2.5, 2}]
```

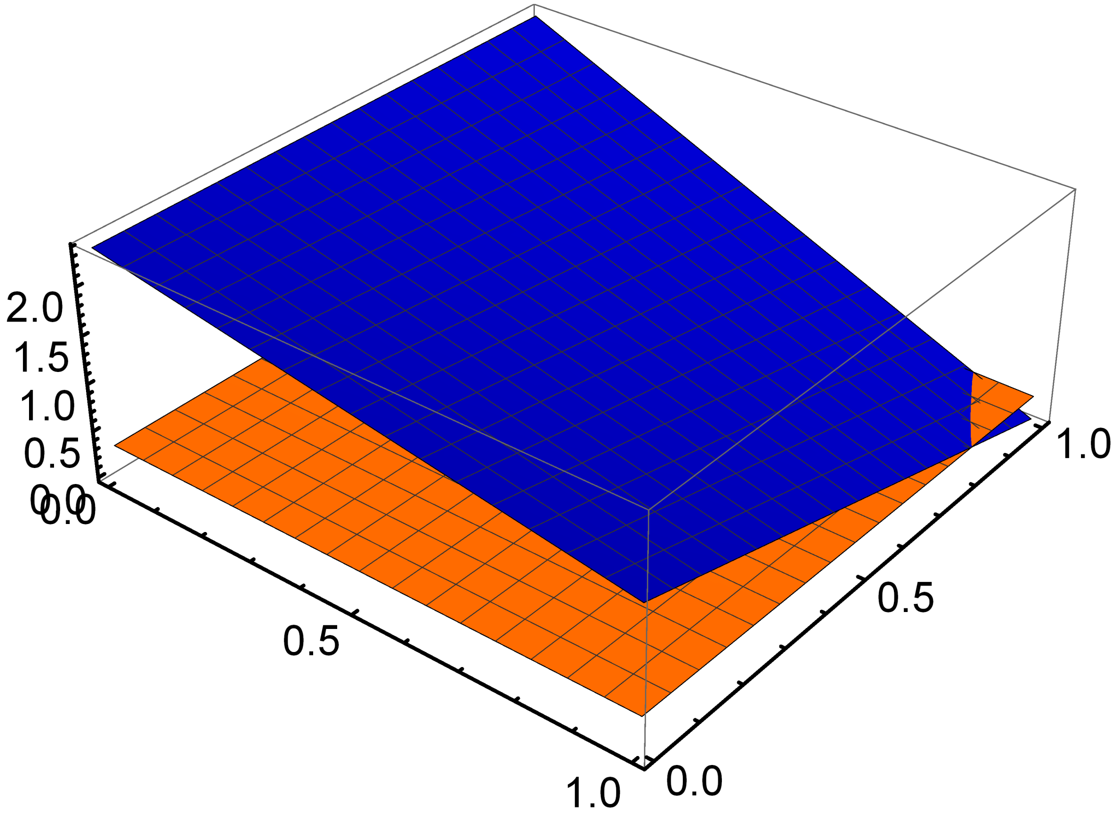

Figure 2: show how the size of the region in which the fitness of helping exceeds that of breeding changes with several model parameters.

Figure 2 a) male lifespan

Plot the region in which the fitness of helping exceeds that of breeding for a male that lives for only one year:

```
wLh1dL = wLh1WEBL /. d → 3 /. L → 0.5
wLb1dL = wLb1WEBL /. d → 3 /. L → 0.5
```

$$0.1675\left(2. - 0.5\,g\,s + \frac{1}{3}\,g\,s\,(-0.25 + 0.25\,g\,s)\right)$$

$$2.38 + g\,(-0.935 - 1.445\,s)$$

```
Reduce[wLh1dL > wLb1dL && 0 < s < 1 && 0 < g < 1]
```

Reduce::ratnz : Reduce was unable to solve the system with inexact coefficients.
The answer was obtained by solving a corresponding exact system and numericizing the result. ≫

$$0.81696 < s < 1.\ \&\&\ -\frac{0.00746269\,(4488. + 6467.\,s)}{s^2} + 7.41815\times10^{-10}\sqrt{\left(\frac{1}{s^4}\left(2.03848\times10^{21} + 5.8747\times10^{21}\,s + 4.49882\times10^{21}\,s^2\right)\right)} < g < 1.$$

$$g1plot = -\frac{1}{s^2}\,0.00746268656716418\grave{}\,\left(4488.\grave{} + 6467.\grave{}\,s\right) + 7.418145030396175\grave{}*\hat{}-10\sqrt{\left(\frac{1}{s^4}\left(2.0384753306070963\grave{}*\hat{}21 + 5.87469695322464\grave{}*\hat{}21\,s + 4.4988204478867057\grave{}*\hat{}21\,s^2\right)\right)};$$

```
Plot1 = Plot[g1plot, {s, 0.8169603491252666, 1}, PlotRange → {{0, 1}, {0, 1}},
  Filling → Top, PlotStyle → Black, LabelStyle → Directive[Black, FontSize → 22],
  ImageSize → Large, Frame → True, FrameStyle → Directive[Thick, Black]]
```

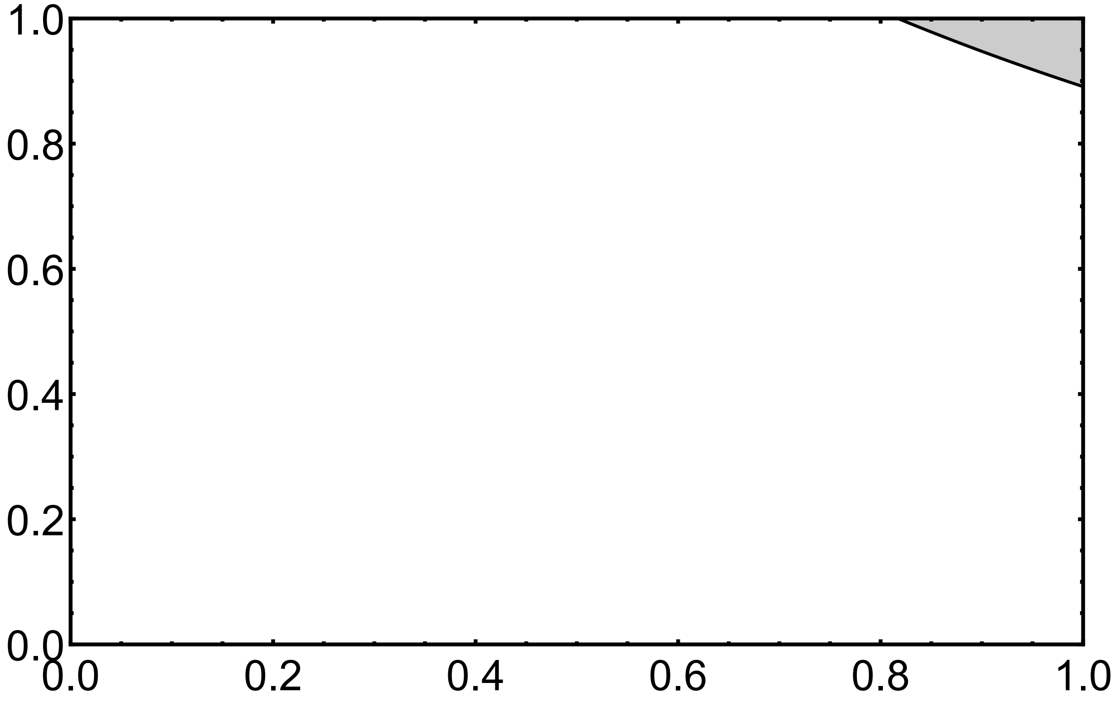

Plot the region in which the fitness of helping in the first year exceeds that of breeding in the first year for a male that lives for 7 adult breeding seasons:

```
wLh7 = Simplify[wh1 + Sum[(1 - mh) * ((1 - mb) ^j) * wbA, {A, 2, 7}]];
wLb7 = Sum[((1 - mb) ^k) * wbA, {A, 1, 7}];
```

```
wLh7dL = Simplify[
   wLh7 /. ro → 0.5 /. ob → 2.89 /. oh → 3.56 /. oe → 1.87 /. mb → 0.5 /. d → 3 /. L → 0.5];
wLb7dL = Simplify[wLb7 /. ro → 0.5 /. ob → 2.89 /. oh → 3.56 /. oe → 1.87 /. mb → 0.5 /.
     d → 3 /. L → 0.5];
```

```
Reduce[wLh7dL > wLb7dL && 0 < s < 1 && 0 < g < 1]
```

Reduce::ratnz : Reduce was unable to solve the system with inexact coefficients.

The answer was obtained by solving a corresponding exact system and numericizing the result. ≫

$$0.110511 < s < 1. \;\&\&\; -\frac{1}{s^2}\, 1.1859\times10^{-19}\left(2.28124\times10^{20} + 3.23042\times10^{20}\, s\right) + 2.89362\times10^{-32}\sqrt{\left(\frac{1}{s^4}\left(8.74089\times10^{65} + 2.47556\times10^{66}\, s + 1.82754\times10^{66}\, s^2\right)\right)} < g < 1.$$

```
g7plot = - 1/s^2 1.1858988724860398`*^-19
      (2.2812399034945153`*^20 + 3.2304178005171424`*^20 s) +
   2.893615412074792`*^-32 Sqrt[(1/s^4 (8.740889173360102`*^65 +
         2.4755593600406207`*^66 s + 1.8275419289691548`*^66 s^2))];

Plot7 = Plot[g7plot, {s, 0.11051063442031395, 1}, PlotRange → {{0, 1}, {0, 1}},
  Filling → Top, PlotStyle → Black, LabelStyle → Directive[Black, FontSize → 22],
  ImageSize → Large, Frame → True, FrameStyle → Directive[Thick, Black]]
```

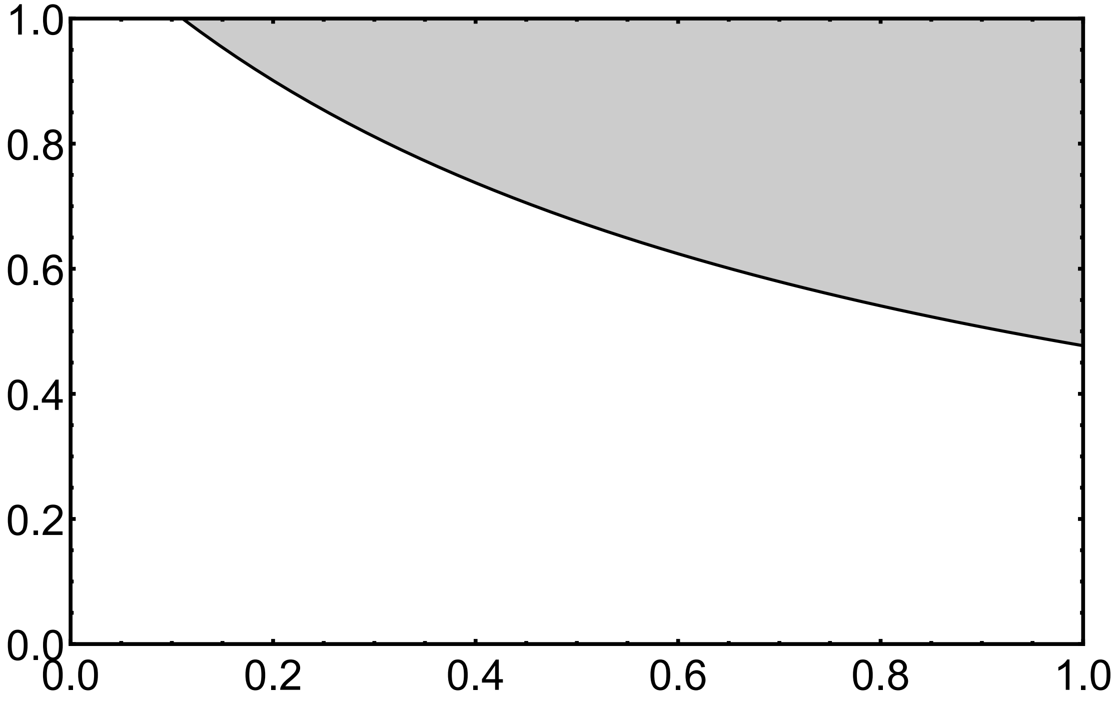

Region in which the fitness of helping in the first year exceeds that of breeding in the first year for a male that lives for 6 adult breeding seasons:

```
wLh6 = Simplify[wh1 + Sum[(1 - mh) * ((1 - mb) ^j) * wbA, {A, 2, 6}]];
wLb6 = Sum[((1 - mb) ^k) * wbA, {A, 1, 6}];

wLh6dL = Simplify[
   wLh6 /. ro → 0.5 /. ob → 2.89 /. oh → 3.56 /. oe → 1.87 /. mb → 0.5 /. d → 3 /. L → 0.5];
wLb6dL = Simplify[wLb6 /. ro → 0.5 /. ob → 2.89 /. oh → 3.56 /. oe → 1.87 /. mb → 0.5 /.
     d → 3 /. L → 0.5];
```

```
Reduce[wLh6dL > wLb6dL && 0 < s < 1 && 0 < g < 1]
```

Reduce::ratnz : Reduce was unable to solve the system with inexact coefficients.
The answer was obtained by solving a corresponding exact system and numericizing the result. ≫

$$0.12668 < s < 1. \;\&\&\; -\frac{1}{s^2}\,0.0000932836\,(290\,411. + 411\,297.\,s) + 5.41528\times10^{-18}\sqrt{\left(\frac{1}{s^4}\left(2.50262\times10^{37} + 7.08871\times10^{37}\,s + 5.23768\times10^{37}\,s^2\right)\right)} < g < 1.$$

```
g6plot = -(1/s^2) 0.00009328358208955225` (290411.` + 411297.` s) +
    5.415281702100986`*^-18 Sqrt[(1/s^4) (2.502617325602629`*^37 +
          7.0887053050220865`*^37 s + 5.237680832763084`*^37 s^2)];
```

```
Plot6 = Plot[g6plot, {s, 0.12667970850210986`, 1}, PlotRange → {{0, 1}, {0, 1}},
  Filling → Top, PlotStyle → Black, LabelStyle → Directive[Black, FontSize → 22],
  ImageSize → Large, Frame → True, FrameStyle → Directive[Thick, Black]]
```

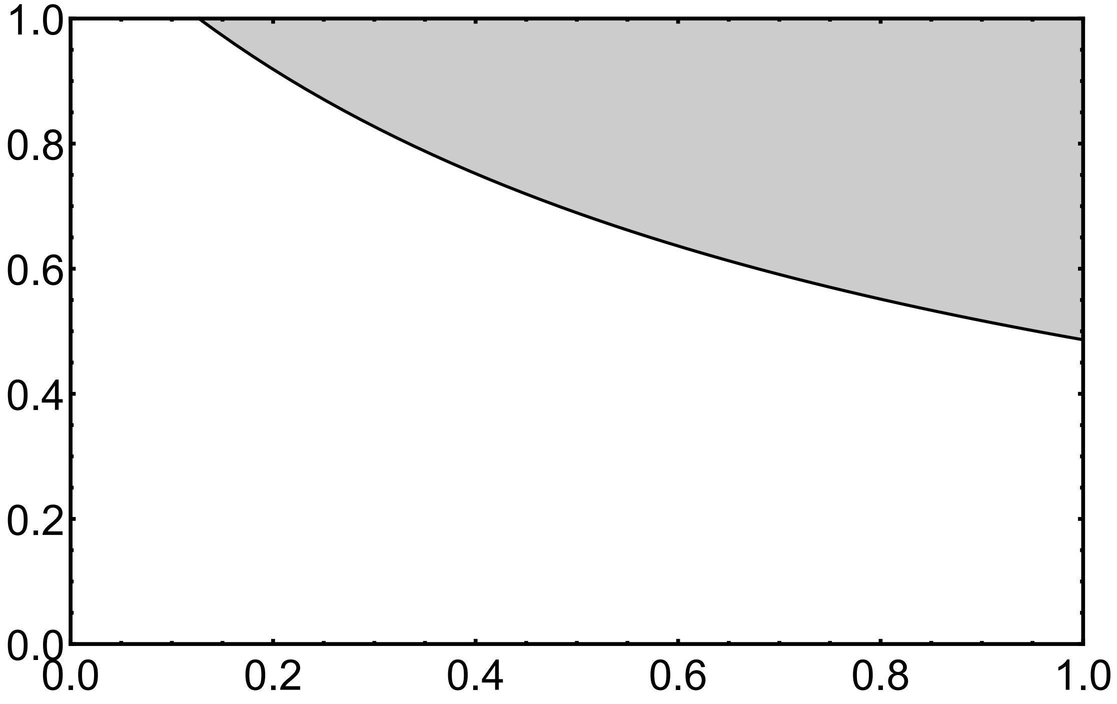

Region in which the fitness of helping in the first year exceeds that of breeding in the first year for a male that lives for 5 adult breeding seasons:

```
wLh5 = Simplify[wh1 + Sum[(1 - mh) * ((1 - mb) ^j) * wbA, {A, 2, 5}]];
wLb5 = Sum[((1 - mb) ^k) * wbA, {A, 1, 5}];
```

```
wLh5dL = Simplify[
   wLh5 /. r_o → 0.5 /. o_b → 2.89 /. o_h → 3.56 /. o_e → 1.87 /. mb → 0.5 /. d → 3 /. L → 0.5];
wLb5dL = Simplify[wLb5 /. r_o → 0.5 /. o_b → 2.89 /. o_h → 3.56 /. o_e → 1.87 /. mb → 0.5 /.
     d → 3 /. L → 0.5];
```

```
Reduce[wLh5dL > wLb5dL && 0 < s < 1 && 0 < g < 1]
```

Reduce::ratnz : Reduce was unable to solve the system with inexact coefficients.
The answer was obtained by solving a corresponding exact system and numericizing the result. >>

$$0.158451 < s < 1. \;\&\&\; -\frac{1}{s^2}\,0.00205224\,(13243. + 18761.\,s) + 1.25061\times10^{-17}\sqrt{\left(\frac{1}{s^4}\left(4.72267\times10^{36} + 1.3381\times10^{37}\,s + 9.90394\times10^{36}\,s^2\right)\right)} < g < 1.$$

$$g5plot = -\frac{1}{s^2}\,0.0020522388059701496`\,\left(13243.` + 18761.`\,s\right) + 1.250605739361558`*^{-17}\sqrt{\left(\frac{1}{s^4}\left(4.7226712413584546`*^{36} + 1.3380961286585513`*^{37}\,s + 9.903935691749722`*^{36}\,s^2\right)\right)};$$

```
Plot5 = Plot[g5plot, {s, 0.1584511094053316, 1}, PlotRange → {{0, 1}, {0, 1}},
  Filling → Top, PlotStyle → Black, LabelStyle → Directive[Black, FontSize → 22],
  ImageSize → Large, Frame → True, FrameStyle → Directive[Thick, Black]]
```

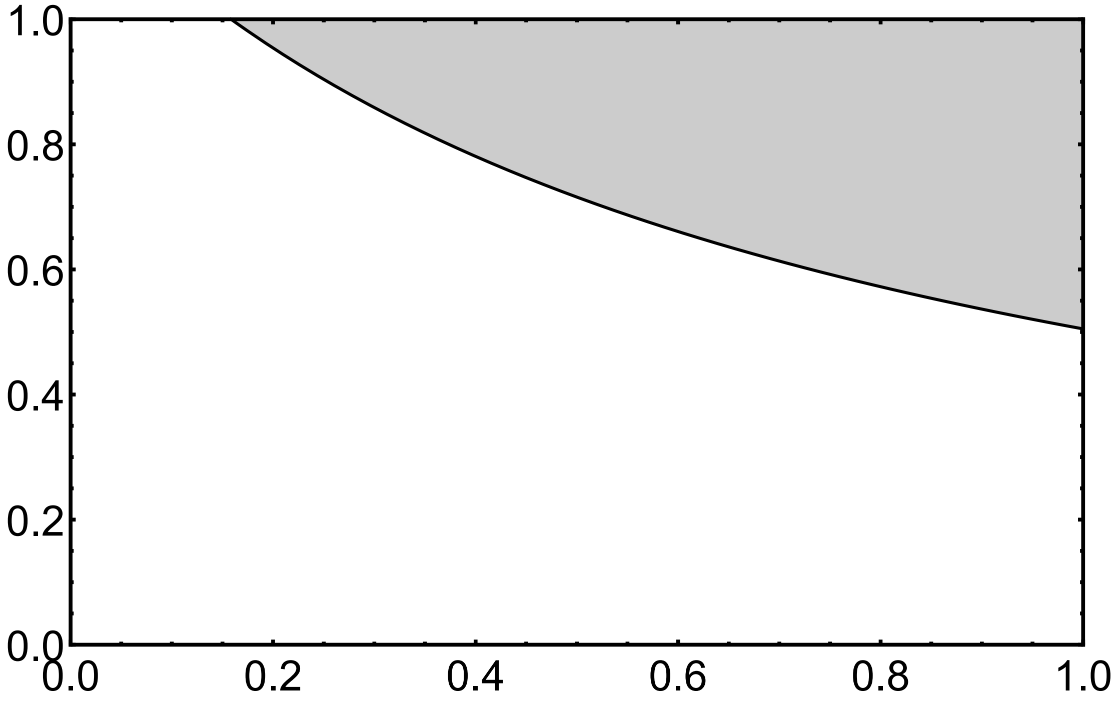

Region in which the fitness of helping in the first year exceeds that of breeding in

the first year for a male that lives for 4 adult breeding seasons:

```
wLh4 = Simplify[wh1 + Sum[(1 - mh) * ((1 - mb) ^j) * wbA, {A, 2, 4}]];
wLb4 = Sum[((1 - mb) ^k) * wbA, {A, 1, 4}];

wLh4dL = Simplify[
   wLh4 /. ro → 0.5 /. ob → 2.89 /. oh → 3.56 /. oe → 1.87 /. mb → 0.5 /. d → 3 /. L → 0.5];
wLb4dL = Simplify[wLb4 /. ro → 0.5 /. ob → 2.89 /. oh → 3.56 /. oe → 1.87 /. mb → 0.5 /.
     d → 3 /. L → 0.5];

Reduce[wLh4dL > wLb4dL && 0 < s < 1 && 0 < g < 1]
```

Reduce::ratnz : Reduce was unable to solve the system with inexact coefficients.
The answer was obtained by solving a corresponding exact system and numericizing the result. ≫

$$0.220058 < s < 1. \&\& -\frac{1}{s^2}\,6.94635\times10^{-15}\left(3.94266\times10^{15}+5.58935\times10^{15}\,s\right)+1.41137\times10^{-27}\sqrt{\left(\frac{1}{s^4}\left(3.7654\times10^{56}+1.06761\times10^{57}\,s+7.92853\times10^{56}\,s^2\right)\right)} < g < 1.$$

```
g4plot =
  -1/s^2 6.946352196178719`*^-15 (3.9426631550208`*^15 + 5.589345113086981`*^15 s) +
   1.411369351948302`*^-27 √((1/s^4) (3.7654040231157253`*^56 +
        1.0676104829599072`*^57 s + 7.92853169963115`*^56 s^2));
```

```
Plot4 = Plot[g4plot, {s, 0.22005823655541415, 1}, PlotRange → {{0, 1}, {0, 1}},
  Filling → Top, PlotStyle → Black, LabelStyle → Directive[Black, FontSize → 22],
  ImageSize → Large, Frame → True, FrameStyle → Directive[Thick, Black]]
```

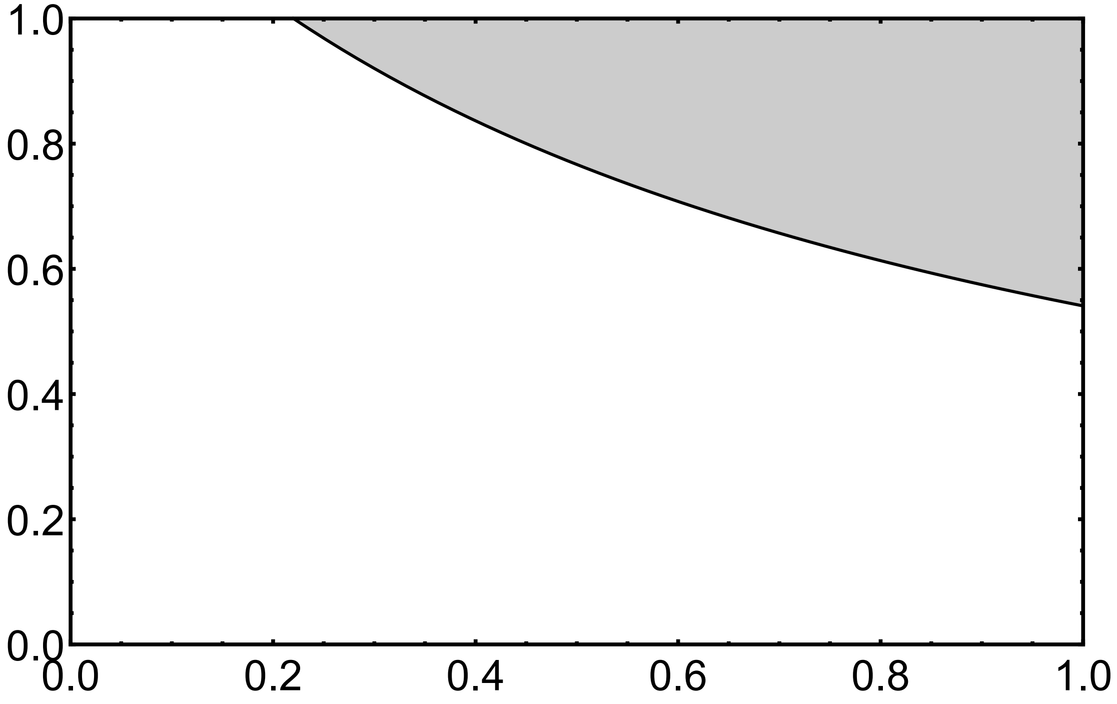

Region in which the fitness of helping in the first year exceeds that of breeding in the first year for a male that lives for 3 adult breeding seasons:

```
wLh3 = Simplify[wh1 + Sum[(1 - mh) * ((1 - mb) ^j) * wbA, {A, 2, 3}]];
wLb3 = Sum[((1 - mb) ^k) * wbA, {A, 1, 3}];

wLh3dL = Simplify[
   wLh3 /. ro → 0.5 /. ob → 2.89 /. oh → 3.56 /. oe → 1.87 /. mb → 0.5 /. d → 3 /. L → 0.5];
wLb3dL = Simplify[wLb3 /. ro → 0.5 /. ob → 2.89 /. oh → 3.56 /. oe → 1.87 /. mb → 0.5 /.
     d → 3 /. L → 0.5];

Reduce[wLh3dL > wLb3dL && 0 < s < 1 && 0 < g < 1]
```

Reduce::ratnz : Reduce was unable to solve the system with inexact coefficients.
The answer was obtained by solving a corresponding exact system and numericizing the result. >>

$$0.335989 < s < 1. \;\&\&\; -\frac{1}{s^2}\, 2.12102\times10^{-16}\left(1.3159\times10^{17} + 1.86864\times10^{17}\, s\right) + 2.07257\times10^{-29}\sqrt{\left(\frac{1}{s^4}\left(1.81348\times10^{60} + 5.15049\times10^{60}\, s + 3.8492\times10^{60}\, s^2\right)\right)} < g < 1.$$

```
g3plot =
  -(1/s^2) 2.12102309183295`*^-16 (1.315895516115024`*^17 + 1.868642001627511`*^17 s) +
   2.0725741209731964`*^-29 Sqrt[(1/s^4 (1.8134829030020376`*^60 +
        5.150485399916481`*^60 s + 3.849197325606228`*^60 s^2))];
```

```
Plot3 = Plot[g3plot, {s, 0.33598879168420076, 1}, PlotRange → {{0, 1}, {0, 1}},
  Filling → Top, PlotStyle → Black, LabelStyle → Directive[Black, FontSize → 22],
  ImageSize → Large, Frame → True, FrameStyle → Directive[Thick, Black]]
```

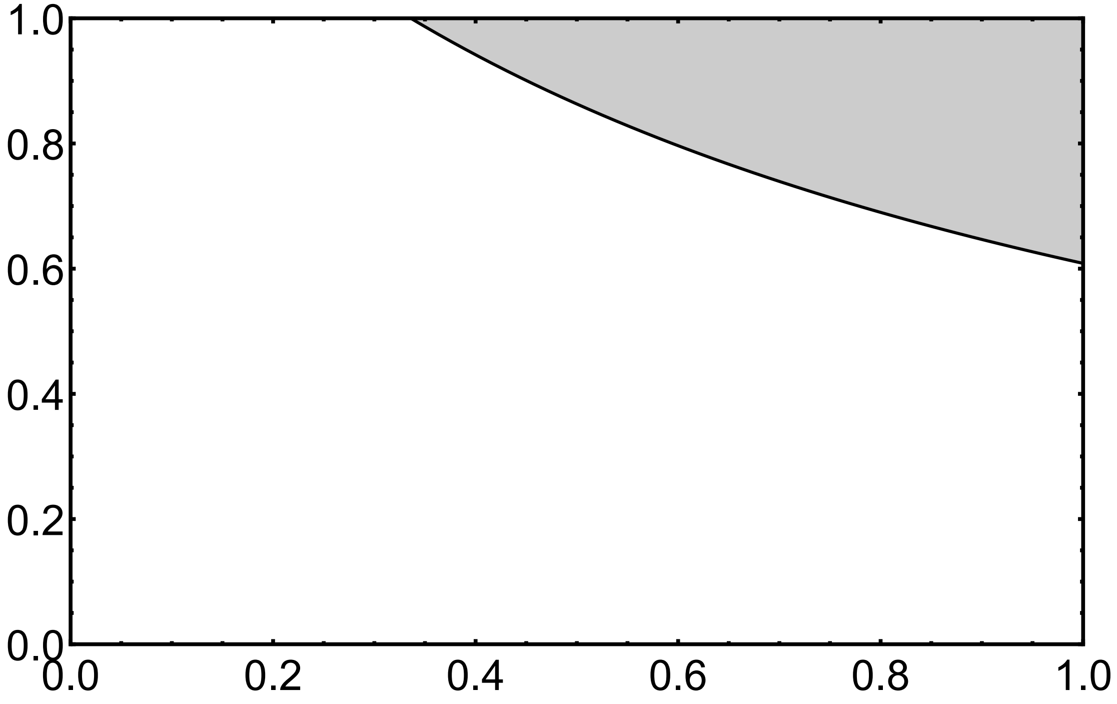

Region in which the fitness of helping in the first year exceeds that of breeding in the first year for a male that lives for 2 adult breeding seasons:

```
wLh2 = Simplify[w_h1 + Sum[(1 - mh) * ((1 - mb) ^j) * w_bA, {A, 2, 2}]];
wLb2 = Sum[((1 - mb) ^k) * w_bA, {A, 1, 2}];
```

```
wLh2dL = Simplify[
   wLh2 /. r_o → 0.5 /. o_b → 2.89 /. o_h → 3.56 /. o_e → 1.87 /. mb → 0.5 /. d → 3 /. L → 0.5];
wLb2dL = Simplify[wLb2 /. r_o → 0.5 /. o_b → 2.89 /. o_h → 3.56 /. o_e → 1.87 /. mb → 0.5 /.
     d → 3 /. L → 0.5];
```

```
Reduce[wLh2dL > wLb2dL && 0 < s < 1 && 0 < g < 1]
```

Reduce::ratnz : Reduce was unable to solve the system with inexact coefficients.
The answer was obtained by solving a corresponding exact system and numericizing the result. >>

$$0.538142 < s < 1. \&\& -\frac{1}{s^2} 4.34038\times 10^{-26} \left(6.75193\times 10^{26} + 9.62842\times 10^{26} s\right) + 3.14959\times 10^{-40} \sqrt{\left(\frac{1}{s^4}\left(8.65774\times 10^{81} + 2.46923\times 10^{82} s + 1.86531\times 10^{82} s^2\right)\right)} < g < 1.$$

```
g2plot =
  -1/s^2 4.340383346254197`*^-26 (6.751931295318684`*^26 + 9.628422524518622`*^26 s) +
   3.1495894008525614`*^-40 Sqrt[(1/s^4 (8.657735730955441`*^81 +
        2.4692294419837268`*^82 s + 1.8653106604949497`*^82 s^2))];
```

```
Plot2 = Plot[g2plot, {s, 0.5381423197372239, 1}, PlotRange -> {{0, 1}, {0, 1}},
  Filling -> Top, PlotStyle -> Black, LabelStyle -> Directive[Black, FontSize -> 22],
  ImageSize -> Large, Frame -> True, FrameStyle -> Directive[Thick, Black]]
```

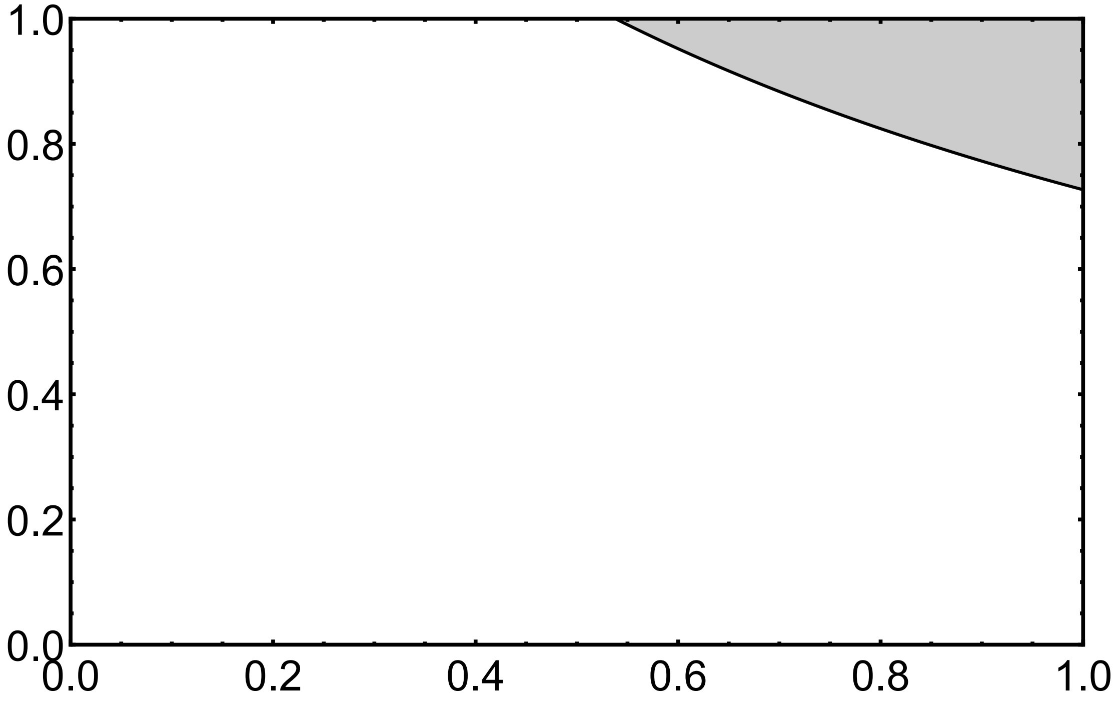

Overlay these plots:

```
PlotAll = Show[Plot1, Plot2, Plot3, Plot4, Plot5, Plot6, Plot7]
```

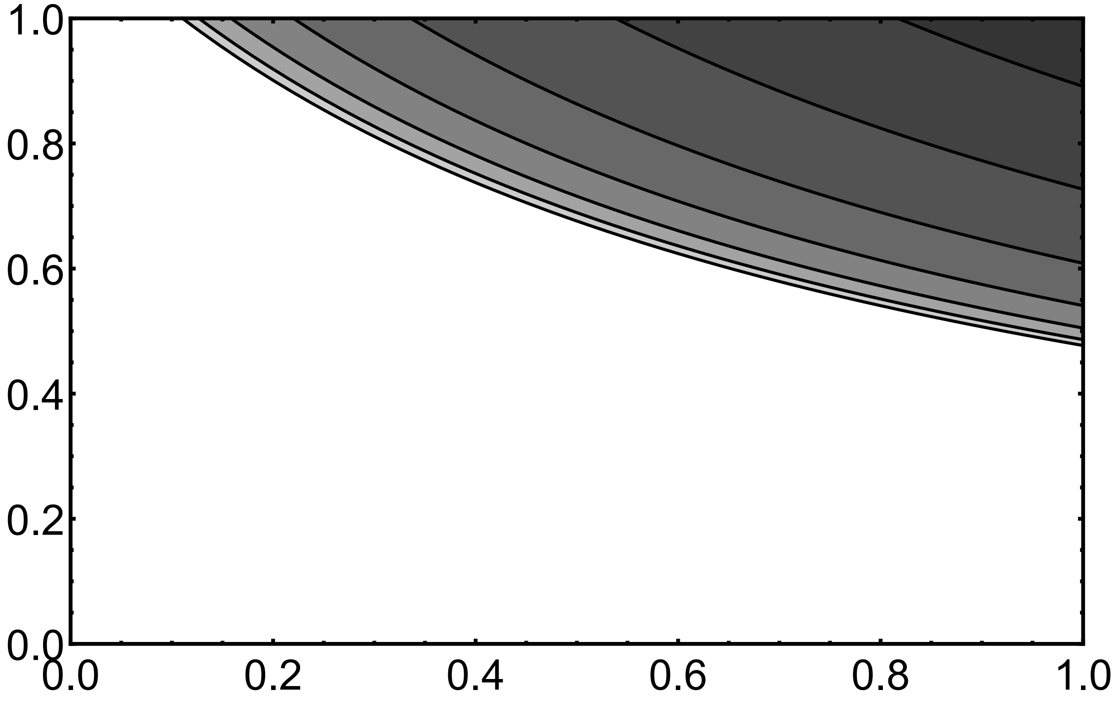

Figure 2 b) annual mortality probability (m_b)

Choose a lifespan of seven adult breeding seasons (8 years).

Vary m_b from 0 to 1.

Use L=0.5

```
wLh7 = Simplify[wh1 + Sum[(1 - mh) * ((1 - mb) ^j) * wbA, {A, 2, 7}]];
wLb7 = Sum[((1 - mb) ^k) * wbA, {A, 1, 7}];
```

m_b = 0.1

```
wLb7dmb01 = Simplify[
   wLh7 /. ro → 0.5 /. ob → 2.89 /. oh → 3.56 /. oe → 1.87 /. mb → 0.1 /. d → 3 /. L → 0.5];
wLb7dmb01 = Simplify[wLb7 /. ro → 0.5 /. ob → 2.89 /. oh → 3.56 /. oe → 1.87 /. mb → 0.1 /.
     d → 3 /. L → 0.5];
```

```
Reduce[wLh7dmb01 > wLb7dmb01 && 0 < s < 1 && 0 < g < 1]
```

Reduce::ratnz : Reduce was unable to solve the system with inexact coefficients.
The answer was obtained by solving a corresponding exact system and numericizing the result. ≫

$$0.489319 < s < 1. \&\& -\frac{1}{s^2} 1.04862\times10^{-15}\left(2.97966\times10^{16}+4.27116\times10^{16}\,s\right)+ 5.15798\times10^{-26}\sqrt{\left(\frac{1}{s^4}\left(3.6695\times10^{53}+1.052\times10^{54}\,s+7.94041\times10^{53}\,s^2\right)\right)} < g < 1.$$

```
g7mb01plot =
  - 1/s^2 1.048615193931737`*^-15 (2.9796610492789724`*^16 + 4.271157177649176`*^16 s) +
   5.157980845381842`*^-26 Sqrt[(1/s^4 (3.6694972779336946`*^53 +
        1.0519988265647338`*^54 s + 7.940405306815431`*^53 s^2))];
```

```
Plot7mb01 = Plot[g7mb01plot, {s, 0.48931930609877194, 1}, PlotRange → {{0, 1}, {0, 1}},
  Filling → Top, PlotStyle → Black, LabelStyle → Directive[Black, FontSize → 22],
  ImageSize → Large, Frame → True, FrameStyle → Directive[Thick, Black]]
```

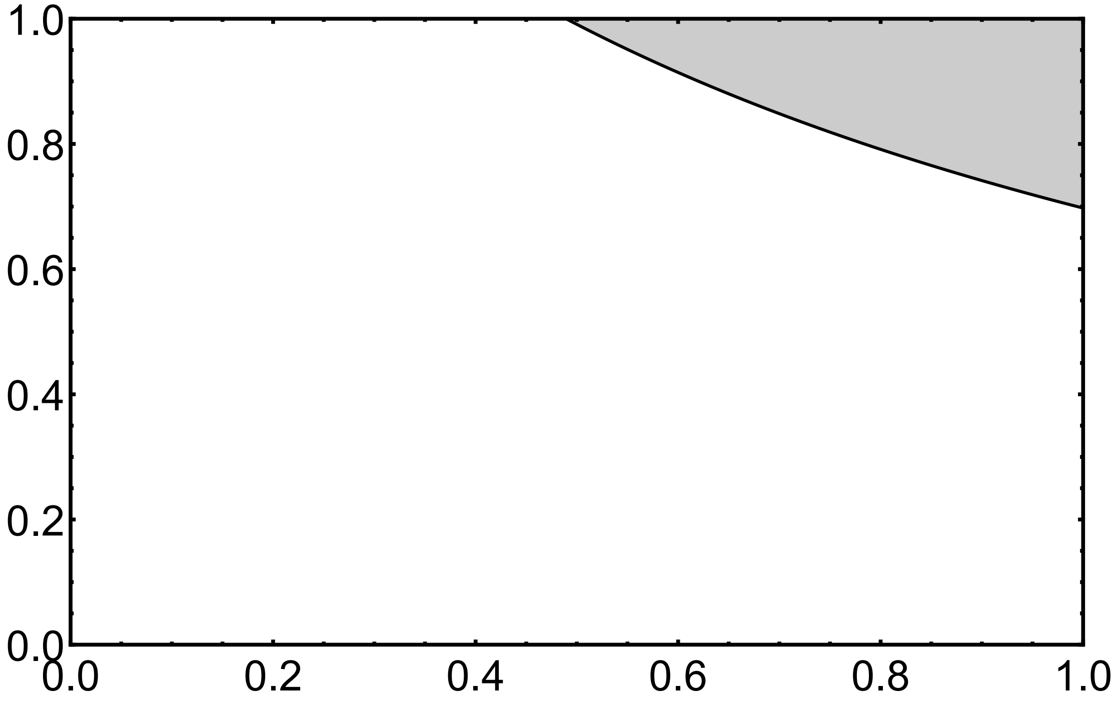

$m_b = 0.3$

```
wLh7dmb03 = Simplify[
   wLh7 /. rₒ → 0.5 /. σb → 2.89 /. σh → 3.56 /. σe → 1.87 /. mb → 0.3 /. d → 3 /. L → 0.5];
wLb7dmb03 = Simplify[wLb7 /. rₒ → 0.5 /. σb → 2.89 /. σh → 3.56 /. σe → 1.87 /. mb → 0.3 /.
     d → 3 /. L → 0.5];
```

```
Reduce[wLh7dmb03 > wLb7dmb03 && 0 < s < 1 && 0 < g < 1]
```

Reduce::ratnz : Reduce was unable to solve the system with inexact coefficients.
The answer was obtained by solving a corresponding exact system and numericizing the result. >>

$$0.174933 < s < 1. \ \&\& -\frac{1}{s^2} 4.58112\times10^{-15}\left(6.22699\times10^{15}+8.85953\times10^{15}\, s\right)+ 4.06469\times10^{-26}\sqrt{\left(\frac{1}{s^4}\left(4.92544\times10^{53}+1.40155\times10^{54}\, s+1.04018\times10^{54}\, s^2\right)\right)} < g < 1.$$

```
g7mb03plot =
  - 1/s^2 4.581124638857875`*^-15 (6.226993270722161`*^15 + 8.85953051964059`*^15 s) +
   4.064694346492379`*^-26 Sqrt[(1/s^4 (4.925442137880895`*^53 +
          1.4015465585437795`*^54 s + 1.0401791419403703`*^54 s^2))];
```

```
Plot7mb03 = Plot[g7mb03plot, {s, 0.17493306493274277, 1}, PlotRange → {{0, 1}, {0, 1}},
  Filling → Top, PlotStyle → Black, LabelStyle → Directive[Black, FontSize → 22],
  ImageSize → Large, Frame → True, FrameStyle → Directive[Thick, Black]]
```

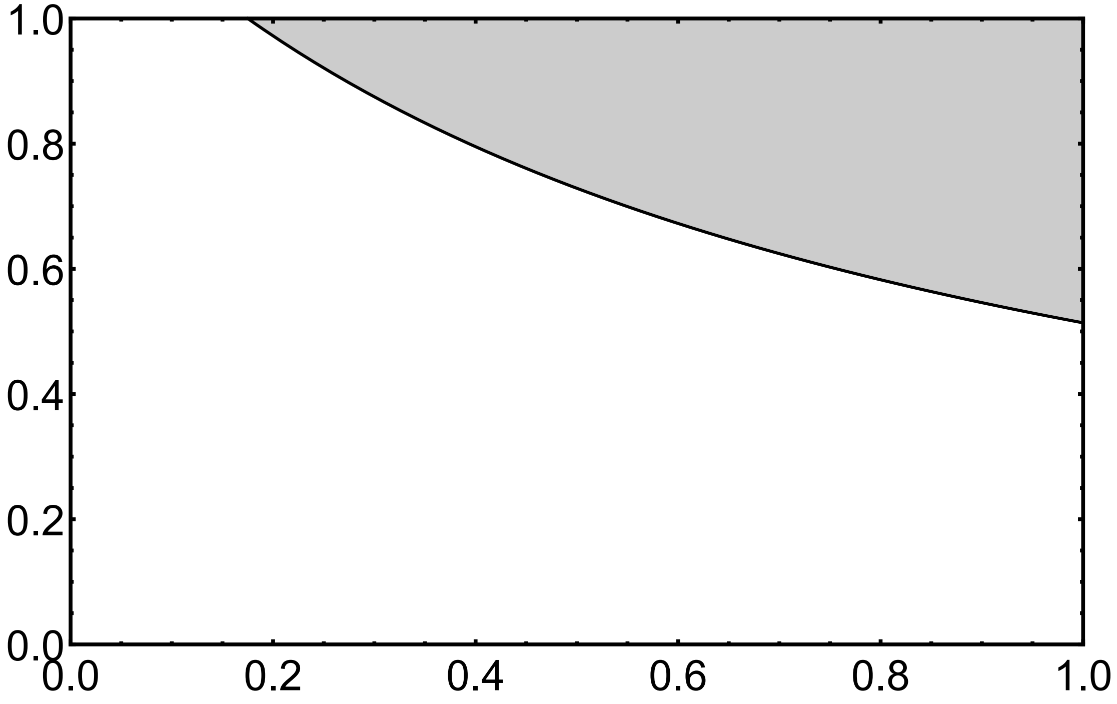

$m_b = 0.5$

```
wLh7dmb05 = Simplify[
   wLh7 /. rₒ → 0.5 /. σb → 2.89 /. σh → 3.56 /. σe → 1.87 /. mb → 0.5 /. d → 3 /. L → 0.5];
wLb7dmb05 = Simplify[wLb7 /. rₒ → 0.5 /. σb → 2.89 /. σh → 3.56 /. σe → 1.87 /. mb → 0.5 /.
     d → 3 /. L → 0.5];
```

```
Reduce[wLh7dmb05 > wLb7dmb05 && 0 < s < 1 && 0 < g < 1]
```

Reduce::ratnz : Reduce was unable to solve the system with inexact coefficients.
The answer was obtained by solving a corresponding exact system and numericizing the result. ≫

$$0.110511 < s < 1. \&\& -\frac{1}{s^2} 1.1859\times10^{-19}\left(2.28124\times10^{20} + 3.23042\times10^{20}\, s\right) + 2.89362\times10^{-32}\sqrt{\left(\frac{1}{s^4}\left(8.74089\times10^{65} + 2.47556\times10^{66}\, s + 1.82754\times10^{66}\, s^2\right)\right)} < g < 1.$$

```
g7mb05plot = - 1/s^2 1.1858988724860398`*^-19
      (2.2812399034945153`*^20 + 3.2304178005171424`*^20 s) +
   2.893615412074792`*^-32 √((1/s^4 (8.740889173360102`*^65 +
         2.4755593600406207`*^66 s + 1.8275419289691548`*^66 s^2)));

Plot7mb05 = Plot[g7mb05plot, {s, 0.11051063442031395, 1}, PlotRange → {{0, 1}, {0, 1}},
  Filling → Top, PlotStyle → Black, LabelStyle → Directive[Black, FontSize → 22],
  ImageSize → Large, Frame → True, FrameStyle → Directive[Thick, Black]]
```

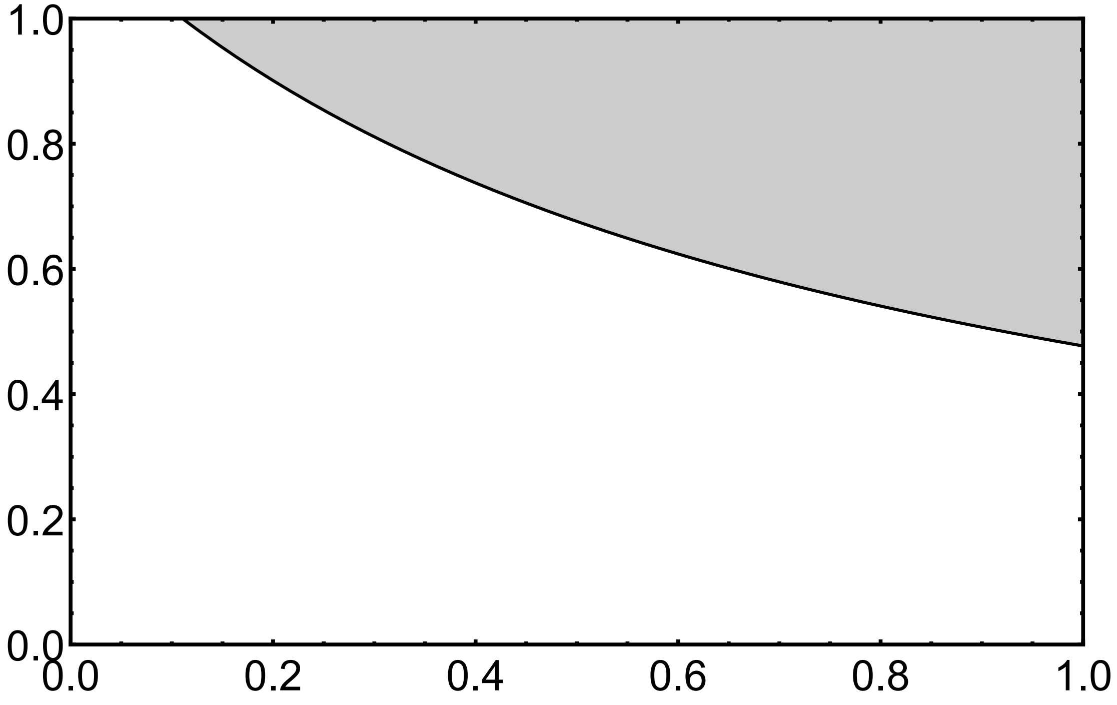

$m_b = 0.7$

```
wLh7dmb07 = Simplify[
   wLh7 /. rₒ → 0.5 /. σb → 2.89 /. σh → 3.56 /. σe → 1.87 /. mb → 0.7 /. d → 3 /. L → 0.5];
wLb7dmb07 = Simplify[wLb7 /. rₒ → 0.5 /. σb → 2.89 /. σh → 3.56 /. σe → 1.87 /. mb → 0.7 /.
     d → 3 /. L → 0.5];
```

```
Reduce[wLh7dmb07 > wLb7dmb07 && 0 < s < 1 && 0 < g < 1]
```

Reduce::ratnz : Reduce was unable to solve the system with inexact coefficients.
The answer was obtained by solving a corresponding exact system and numericizing the result. >>

$$0.123136 < s < 1. \&\& -\frac{1}{s^2} 1.1016\times10^{-13} \left(2.37038\times10^{14} + 3.34559\times10^{14} s\right) + 1.08247\times10^{-25} \sqrt{\left(\frac{1}{s^4}\left(5.81906\times10^{52} + 1.64262\times10^{53} s + 1.21154\times10^{53} s^2\right)\right)} < g < 1.$$

```
g7mb07plot =
  -1/s^2 1.1016023430176242`*^-13 (2.37037942494321`*^14 + 3.34559465202928`*^14 s) +
   1.0824714817640454`*^-25 Sqrt[(1/s^4 (5.819055676508432`*^52 +
         1.642623231228364`*^53 s + 1.2115421782867514`*^53 s^2))];
```

```
Plot7mb07 = Plot[g7mb07plot, {s, 0.12313622984930257, 1}, PlotRange → {{0, 1}, {0, 1}},
  Filling → Top, PlotStyle → Black, LabelStyle → Directive[Black, FontSize → 22],
  ImageSize → Large, Frame → True, FrameStyle → Directive[Thick, Black]]
```

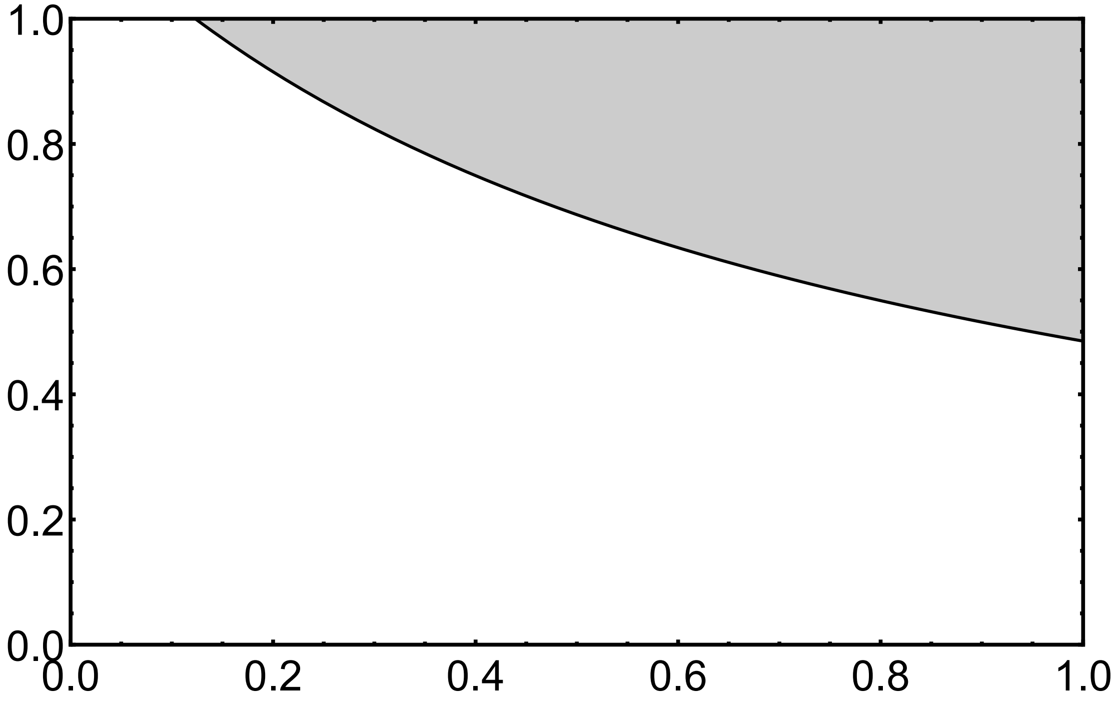

Overlay these plots:

```
PlotAllmb = Show[Plot7mb07, Plot7mb05, Plot7mb03, Plot7mb01]
```

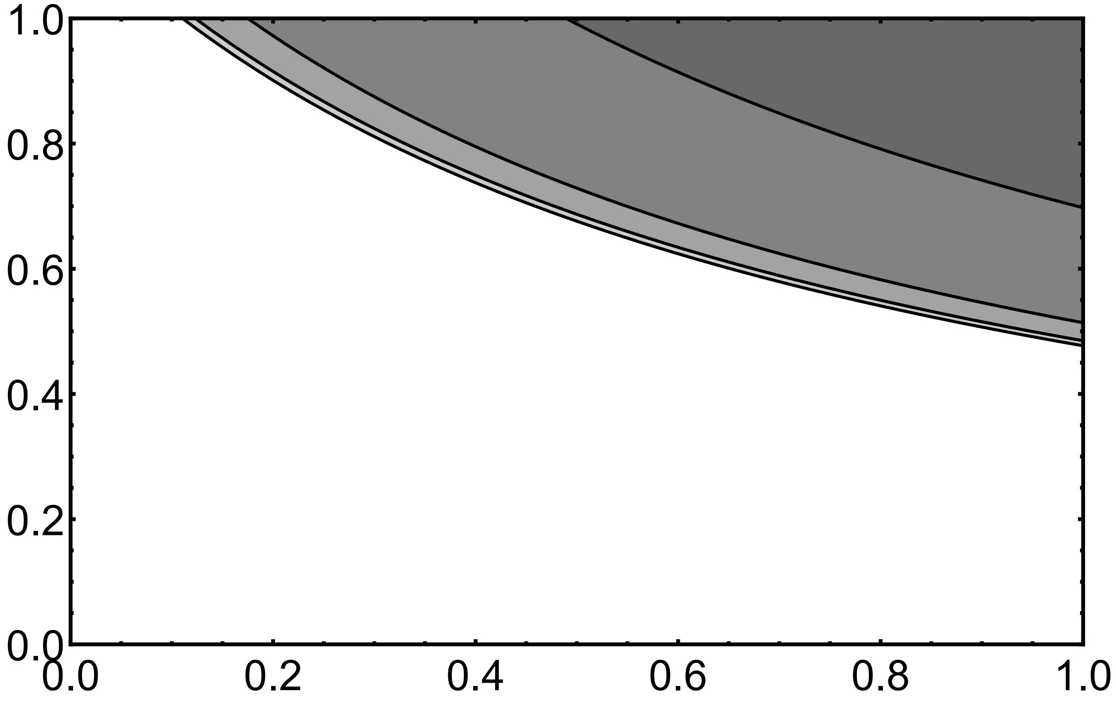

This plot shows the influence of the value of m_b on the size of the region in which helping is favored over breeding, with all parameters drawn from the western bluebird population. The x-axis is s and the y-axis is g. The top line represents m_b=0.1, the bottom line m_b=0.7. The larger m_b is, the larger the region in which helping is favored over breeding; however, this effect attenuates as m_b increases.

Figure 2 c) proportion of annual mortality probability suffered by adults in a group with a helper (L)

Choose a lifespan of seven adult breeding seasons (8 years).
Vary L from 0 to 1.

```
wLh7 = Simplify[wh1 + Sum[(1 - mh) * ((1 - mb) ^j) * wbA, {A, 2, 7}]];
wLb7 = Sum[((1 - mb) ^k) * wbA, {A, 1, 7}];
```

L = 0.25

```
wLh7dL025 = Simplify[
   wLh7 /. ro → 0.5 /. ob → 2.89 /. oh → 3.56 /. oe → 1.87 /. mb → 0.5 /. d → 3 /. L → 0.25];
wLb7dL025 = Simplify[wLb7 /. ro → 0.5 /. ob → 2.89 /. oh → 3.56 /. oe → 1.87 /. mb → 0.5 /.
    d → 3 /. L → 0.25];
```

```
Reduce[wLh7dL025 > wLb7dL025 && 0 < s < 1 && 0 < g < 1]
```

Reduce::ratnz : Reduce was unable to solve the system with inexact coefficients.
The answer was obtained by solving a corresponding exact system and numericizing the result. >>

$$0 < s < 1. \;\&\&\; -\frac{1}{s^2} 3.82916\times10^{-19} \left(6.22422\times10^{19} + 8.70521\times10^{19}\, s\right) + 5.13356\times10^{-32} \sqrt{\left(\frac{1}{s^4}\left(2.15546\times10^{65} + 6.02926\times10^{65}\, s + 4.29453\times10^{65}\, s^2\right)\right)} < g < 1.$$

```
g7L025plot =
  -(1/s^2) 3.8291580001468264`*^-19 (6.2242218220648825`*^19 + 8.70521284135524`*^19 s) +
   5.133558197110475`*^-32 Sqrt[((1/s^4) (2.155458758045453`*^65 +
         6.029260458883849`*^65 s + 4.294532773292547`*^65 s^2))];

Plot7L025 = Plot[g7L025plot, {s, 0, 1}, PlotRange → {{0, 1}, {0, 1}},
  Filling → Top, PlotStyle → Black, LabelStyle → Directive[Black, FontSize → 22],
  ImageSize → Large, Frame → True, FrameStyle → Directive[Thick, Black]]
```

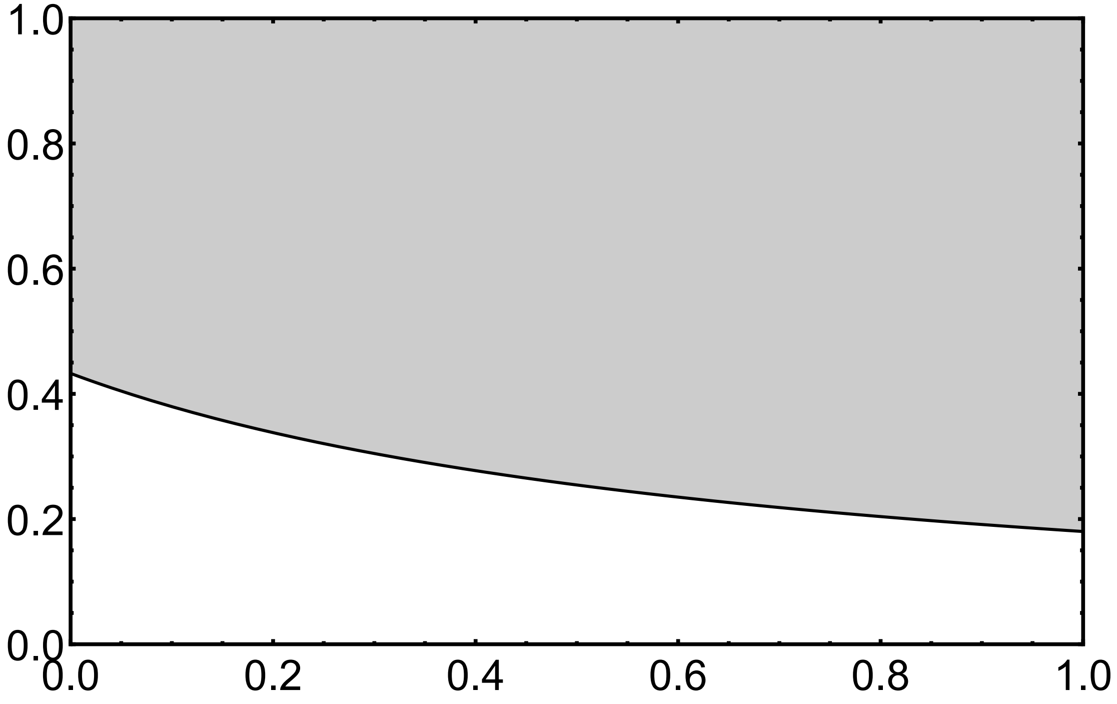

L = 0.5

```
wLh7dL05 = Simplify[
   wLh7 /. r_o → 0.5 /. o_b → 2.89 /. o_h → 3.56 /. o_e → 1.87 /. mb → 0.5 /. d → 3 /. L → 0.5];
wLb7dL05 = Simplify[wLb7 /. r_o → 0.5 /. o_b → 2.89 /. o_h → 3.56 /. o_e → 1.87 /. mb → 0.5 /.
     d → 3 /. L → 0.5];
```

```
Reduce[wLh7dL05 > wLb7dL05 && 0 < s < 1 && 0 < g < 1]
```

Reduce::ratnz : Reduce was unable to solve the system with inexact coefficients.
The answer was obtained by solving a corresponding exact system and numericizing the result. ≫

$$0.110511 < s < 1. \; \&\& \; -\frac{1}{s^2} 1.1859 \times 10^{-19} \left(2.28124 \times 10^{20} + 3.23042 \times 10^{20} s\right) + 2.89362 \times 10^{-32} \sqrt{\left(\frac{1}{s^4} \left(8.74089 \times 10^{65} + 2.47556 \times 10^{66} s + 1.82754 \times 10^{66} s^2\right)\right)} < g < 1.$$

$$\text{g7L05plot} = -\frac{1}{s^2} 1.1858988724860398\text{\`}*\text{\^{}}-19 \left(2.2812399034945153\text{\`}*\text{\^{}}20 + 3.2304178005171424\text{\`}*\text{\^{}}20\, s\right) + 2.893615412074792\text{\`}*\text{\^{}}-32 \sqrt{\left(\frac{1}{s^4}\left(8.740889173360102\text{\`}*\text{\^{}}65 + 2.4755593600406207\text{\`}*\text{\^{}}66\, s + 1.8275419289691548\text{\`}*\text{\^{}}66\, s^2\right)\right)};$$

```
Plot7L05 = Plot[g7L05plot, {s, 0.11051063442031395, 1}, PlotRange → {{0, 1}, {0, 1}},
  Filling → Top, PlotStyle → Black, LabelStyle → Directive[Black, FontSize → 22],
  ImageSize → Large, Frame → True, FrameStyle → Directive[Thick, Black]]
```

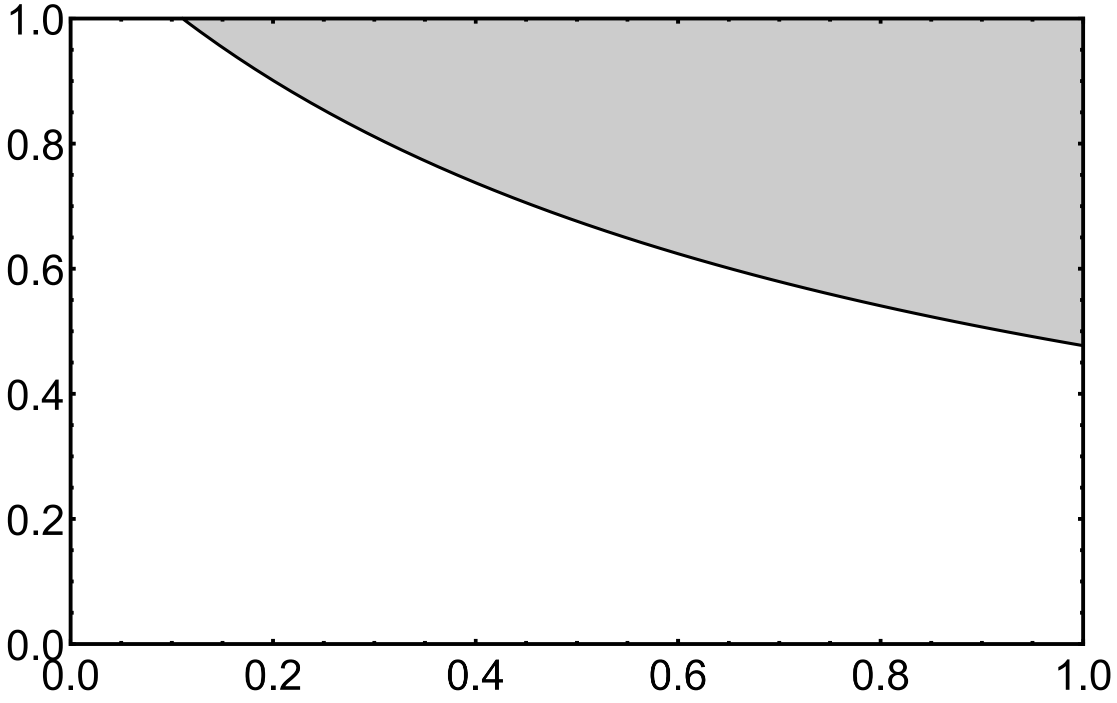

L = 0.75

```
wLh7dL075 = Simplify[
   wLh7 /. ro → 0.5 /. σb → 2.89 /. σh → 3.56 /. σe → 1.87 /. mb → 0.5 /. d → 3 /. L → 0.75];
wLb7dL075 = Simplify[wLb7 /. ro → 0.5 /. σb → 2.89 /. σh → 3.56 /. σe → 1.87 /. mb → 0.5 /.
     d → 3 /. L → 0.75];
```

```
Reduce[wLh7dL075 > wLb7dL075 && 0 < s < 1 && 0 < g < 1]
```

Reduce::ratnz : Reduce was unable to solve the system with inexact coefficients.
The answer was obtained by solving a corresponding exact system and numericizing the result. ≫

$$0.505316 < s < 1. \;\&\&\; -\frac{1}{s^2} 1.17078 \times 10^{-18} \left(2.58569 \times 10^{19} + 3.69713 \times 10^{19}\, s\right) + 9.15232 \times 10^{-31} \sqrt{\left(\frac{1}{s^4}\left(1.09407 \times 10^{63} + 3.12869 \times 10^{63}\, s + 2.36157 \times 10^{63}\, s^2\right)\right)} < g < 1.$$

```
g7L075plot = - (1/s^2) 1.1707831011473888`*^-18
     (2.5856939491328504`*^19 + 3.6971272568732434`*^19 s) +
   9.152318123043537`*^-31 Sqrt[((1/s^4) (1.094069508101944`*^63 +
        3.1286875236523012`*^63 s + 2.3615700157934592`*^63 s^2))];
```

```
Plot7L075 = Plot[g7L075plot, {s, 0.5053161258830751, 1}, PlotRange → {{0, 1}, {0, 1}},
  Filling → Top, PlotStyle → Black, LabelStyle → Directive[Black, FontSize → 22],
  ImageSize → Large, Frame → True, FrameStyle → Directive[Thick, Black]]
```

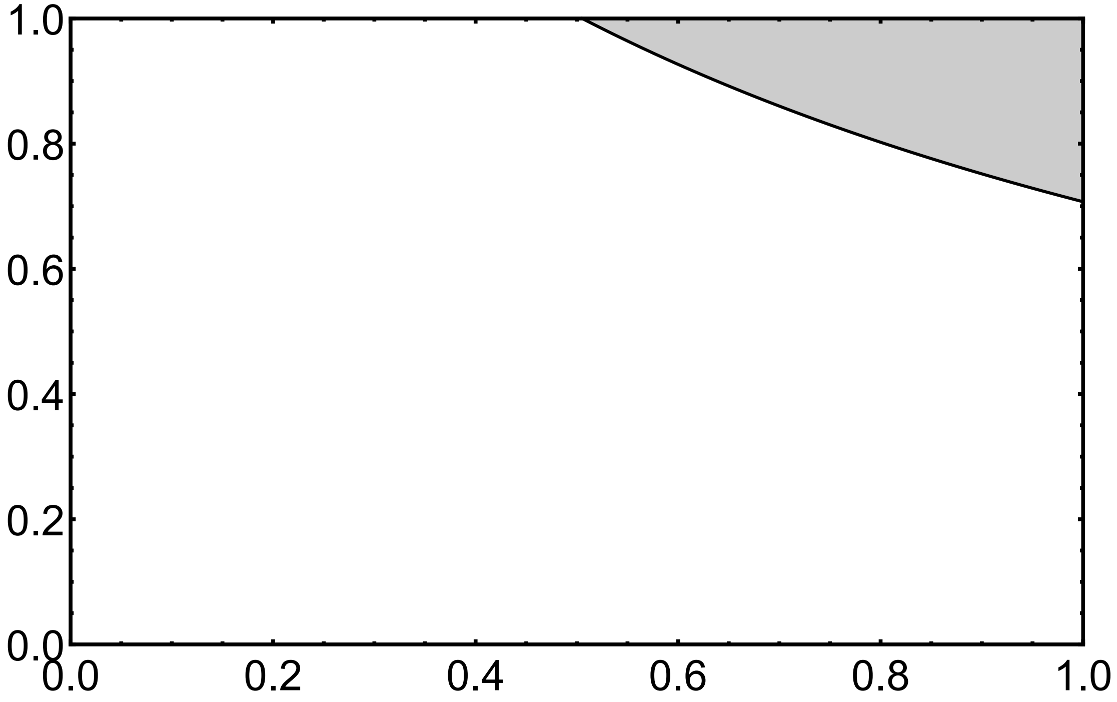

L = 1.0

```
wLh7dL1 = Simplify[
   wLh7 /. rₒ → 0.5 /. σb → 2.89 /. σh → 3.56 /. σe → 1.87 /. mb → 0.5 /. d → 3 /. L → 1];
wLb7dL1 = Simplify[wLb7 /. rₒ → 0.5 /. σb → 2.89 /. σh → 3.56 /. σe → 1.87 /. mb → 0.5 /.
     d → 3 /. L → 1];

Reduce[wLh7dL1 > wLb7dL1 && 0 < s < 1 && 0 < g < 1]
```

Reduce::ratnz : Reduce was unable to solve the system with inexact coefficients.
The answer was obtained by solving a corresponding exact system and numericizing the result. >>

$$0.81696 < s < 1. \ \&\& -\frac{1}{s^2} 3.13954\times10^{-24}\left(1.0668\times10^{25}+1.5372\times10^{25}\,s\right)+ 1.39567\times10^{-31}\sqrt{\left(\frac{1}{s^4}\left(5.75881\times10^{64}+1.65964\times10^{65}\,s+1.27094\times10^{65}\,s^2\right)\right)} < g < 1.$$

```
g7L1plot = - 1/s^2 3.1395439753996684`*^-24
      (1.0667962473489228`*^25 + 1.5372039508924874`*^25 s) +
   1.3956666038800457`*^-31 Sqrt[(1/s^4 (5.758809686775978`*^64 +
         1.6596355723877117`*^65 s + 1.2709425709184305`*^65 s^2))];

Plot7L1 = Plot[g7L1plot, {s, 0.8169603491252743, 1}, PlotRange → {{0, 1}, {0, 1}},
  Filling → Top, PlotStyle → Black, LabelStyle → Directive[Black, FontSize → 22],
  ImageSize → Large, Frame → True, FrameStyle → Directive[Thick, Black]]
```

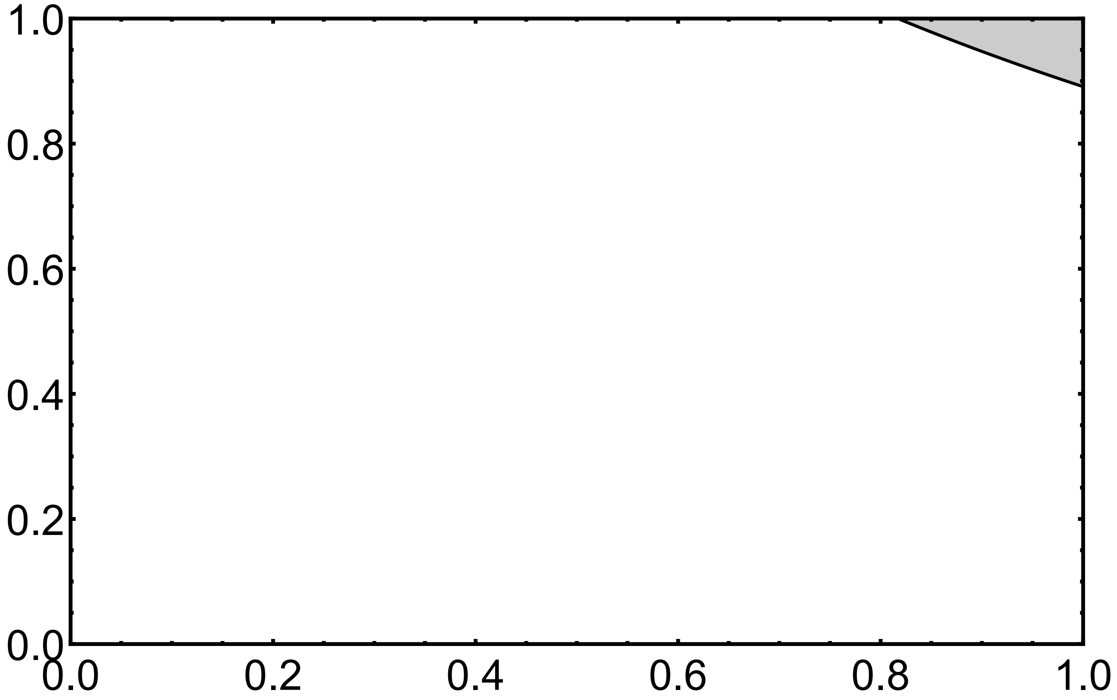

Overlay these plots:

```
PlotAllL = Show[Plot7L025, Plot7L05, Plot7L075, Plot7L1]
```

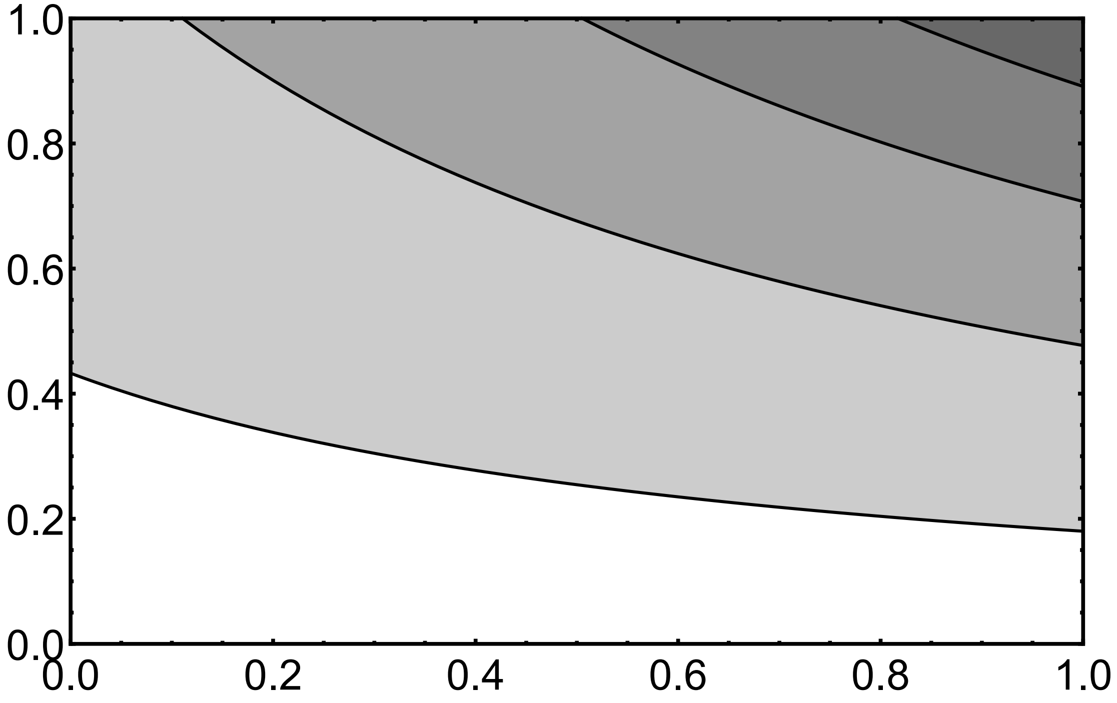

This plot shows the influence of the value of L on the size of the region in which helping is favored over breeding, with all parameters drawn from the western bluebird population. The x-axis is s and the y-axis is g. The top line represents L=1, the bottom line L=0.25. The smaller L is, the larger the region in which helping is favored over breeding.

Figure 2 d) age difference between father and son

Choose a lifespan of seven adult breeding seasons (8 years).

Vary d from 1 to 6.

Use mb=0.5 and L=0.5.

```
wLh7 = Simplify[wh1 + Sum[(1 - mh) * ((1 - mb) ^j) * wbA, {A, 2, 7}]];
wLb7 = Sum[((1 - mb) ^k) * wbA, {A, 1, 7}];
```

d = 1

```
wLh7d1 = Simplify[
   wLh7 /. rₒ → 0.5 /. σb → 2.89 /. σh → 3.56 /. σe → 1.87 /. mb → 0.5 /. d → 1 /. L → 0.5];
wLb7d1 = Simplify[wLb7 /. rₒ → 0.5 /. σb → 2.89 /. σh → 3.56 /. σe → 1.87 /. mb → 0.5 /.
     d → 1 /. L → 0.5];
```

```
Reduce[wLh7d1 > wLb7d1 && 0 < s < 1 && 0 < g < 1]
```

Reduce::ratnz : Reduce was unable to solve the system with inexact coefficients.
The answer was obtained by solving a corresponding exact system and numericizing the result. ≫

$$0.12773 < s < 1. \;\&\&\; -\frac{1}{s^2}\,5.56631\times10^{-20}\left(8.10028\times10^{19} + 9.82383\times10^{19}\,s\right) + 3.14052\times10^{-26}\sqrt{\left(\frac{1}{s^4}\left(2.06125\times10^{52} + 4.99968\times10^{52}\,s + 4.08935\times10^{52}\,s^2\right)\right)} < g < 1.$$

```
g7d1plot =
  - 1/s^2 5.566311401261847`*^-20 (8.100276929083982`*^19 + 9.823827039657243`*^19 s) +
   3.1405203289597995`*^-26 Sqrt[(1/s^4 (2.0612540069241916`*^52 +
          4.999681622271058`*^52 s + 4.089348767057339`*^52 s^2))];
```

```
Plot7d1 = Plot[g7d1plot, {s, 0.12773033827025468, 1}, PlotRange → {{0, 1}, {0, 1}},
  Filling → Top, PlotStyle → Black, LabelStyle → Directive[Black, FontSize → 22],
  ImageSize → Large, Frame → True, FrameStyle → Directive[Thick, Black]]
```

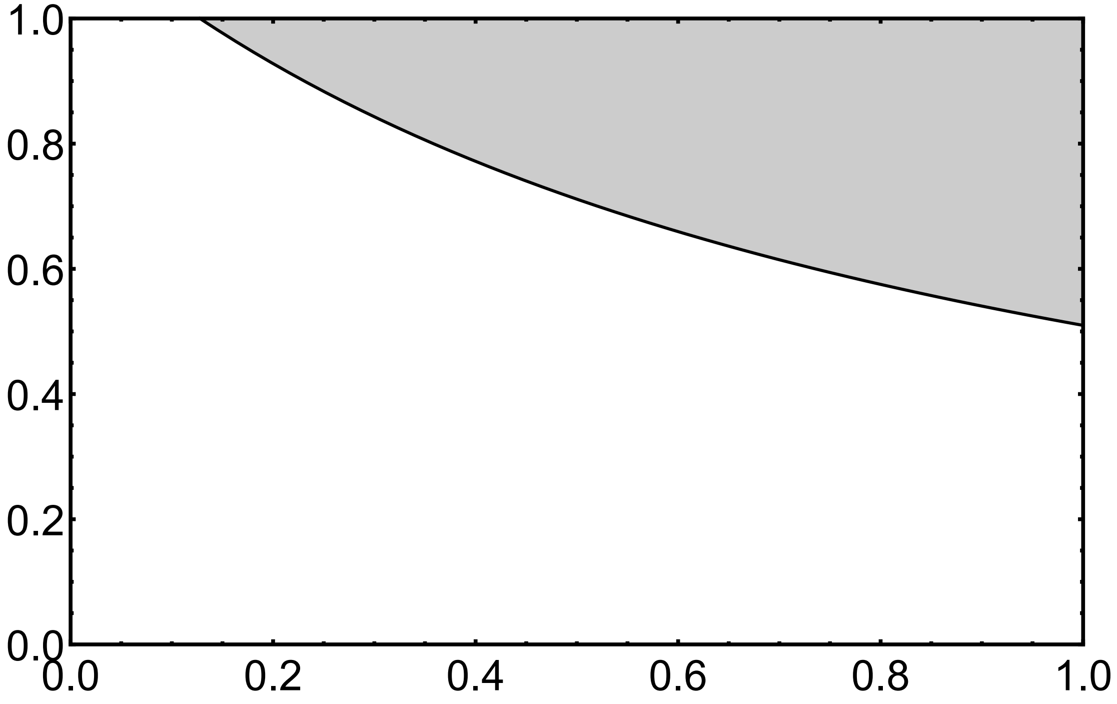

d = 3

```
wLh7d3 = Simplify[
   wLh7 /. rₒ → 0.5 /. σb → 2.89 /. σh → 3.56 /. σe → 1.87 /. mb → 0.5 /. d → 3 /. L → 0.5];
wLb7d3 = Simplify[wLb7 /. rₒ → 0.5 /. σb → 2.89 /. σh → 3.56 /. σe → 1.87 /. mb → 0.5 /.
     d → 3 /. L → 0.5];
```

```
Reduce[wLh7d3 > wLb7d3 && 0 < s < 1 && 0 < g < 1]
```

Reduce::ratnz : Reduce was unable to solve the system with inexact coefficients.
The answer was obtained by solving a corresponding exact system and numericizing the result. ≫

$$0.110511 < s < 1. \;\&\&\; -\frac{1}{s^2}\, 1.1859\times10^{-19}\left(2.28124\times10^{20}+3.23042\times10^{20}\, s\right) + 2.89362\times10^{-32}\sqrt{\left(\frac{1}{s^4}\left(8.74089\times10^{65}+2.47556\times10^{66}\, s+1.82754\times10^{66}\, s^2\right)\right)} < g < 1.$$

```
g7d3plot = -1/s^2 1.1858988724860398`*^-19
    (2.2812399034945153`*^20 + 3.2304178005171424`*^20 s) +
  2.893615412074792`*^-32 Sqrt[(1/s^4 (8.740889173360102`*^65 +
       2.4755593600406207`*^66 s + 1.8275419289691548`*^66 s^2))];
```

```
Plot7d3 = Plot[g7d3plot, {s, 0.11051063442031395, 1}, PlotRange → {{0, 1}, {0, 1}},
  Filling → Top, PlotStyle → Black, LabelStyle → Directive[Black, FontSize → 22],
  ImageSize → Large, Frame → True, FrameStyle → Directive[Thick, Black]]
```

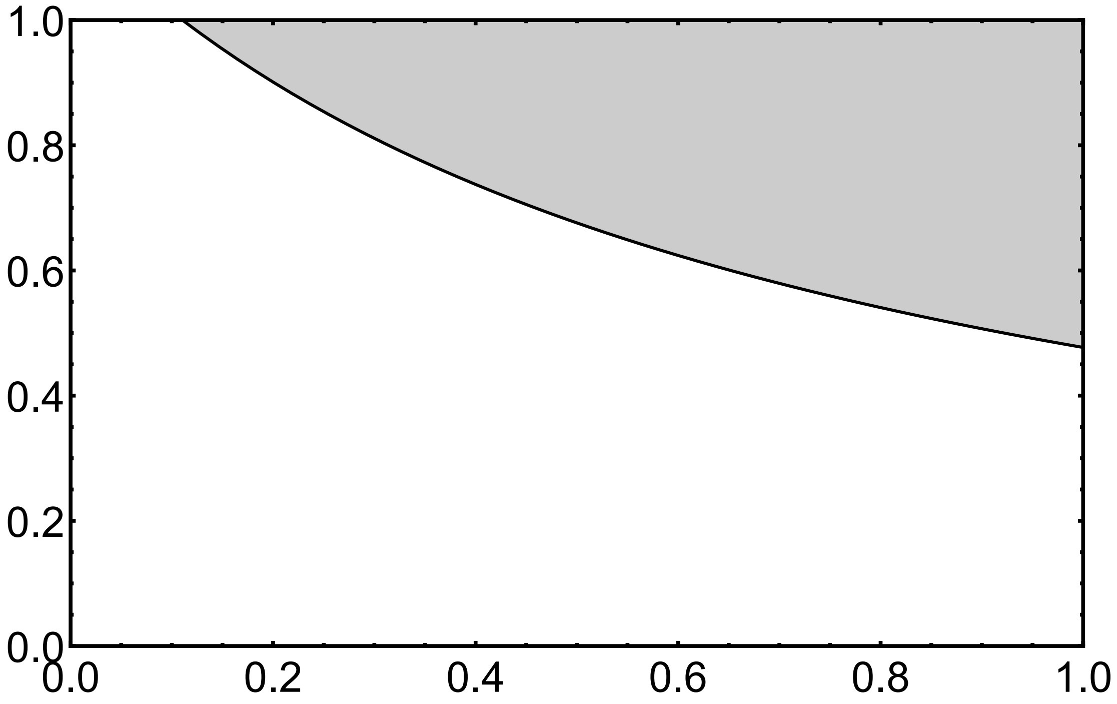

d = 6

```
wLh7d6 = Simplify[
   wLh7 /. rₒ → 0.5 /. ob → 2.89 /. oh → 3.56 /. oe → 1.87 /. mb → 0.5 /. d → 6 /. L → 0.5];
wLb7d6 = Simplify[wLb7 /. rₒ → 0.5 /. ob → 2.89 /. oh → 3.56 /. oe → 1.87 /. mb → 0.5 /.
     d → 6 /. L → 0.5];
```

```
Reduce[wLh7d6 > wLb7d6 && 0 < s < 1 && 0 < g < 1]
```

Reduce::ratnz : Reduce was unable to solve the system with inexact coefficients.
The answer was obtained by solving a corresponding exact system and numericizing the result. ≫

$$0.106079 < s < 1. \&\& -\frac{1}{s^2} 1.01324\times10^{-18}\left(9.34488\times10^{19}+1.38006\times10^{20}\, s\right) + 2.70389\times10^{-31}\sqrt{\left(\frac{1}{s^4}\left(1.22629\times10^{65}+3.622\times10^{65}\, s+2.70446\times10^{65}\, s^2\right)\right)} < g < 1.$$

```
g7d6plot =
  - 1/s^2 1.0132410319346798`*^-18 (9.344883502107807`*^19 + 1.3800586883875671`*^20 s) +
   1.5965229207118505`*^-31 Sqrt[(1/s^4 (3.5174100851970463`*^65 +
         1.0389069799753693`*^66 s + 7.757263789815242`*^65 s^2))];

Plot7d6 = Plot[g7d6plot, {s, 0.10607899419342223, 1}, PlotRange → {{0, 1}, {0, 1}},
  Filling → Top, PlotStyle → Black, LabelStyle → Directive[Black, FontSize → 22],
  ImageSize → Large, Frame → True, FrameStyle → Directive[Thick, Black]]
```

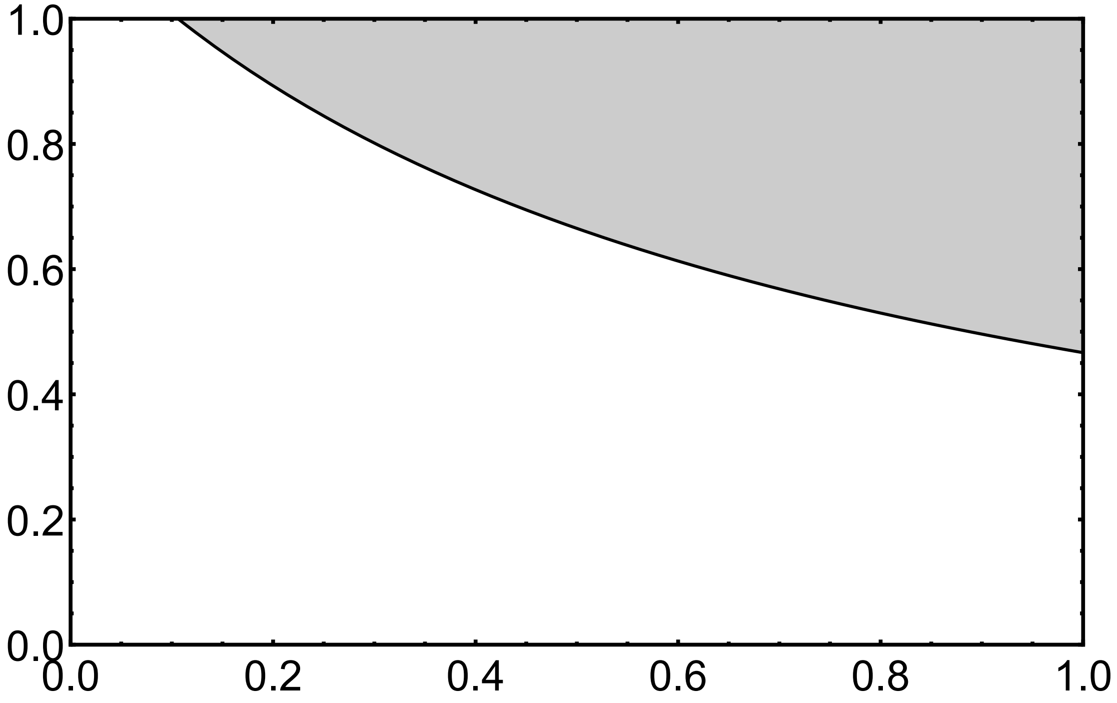

Overlay these plots:

```
PlotAlld = Show[Plot7d1, Plot7d3, Plot7d6]
```

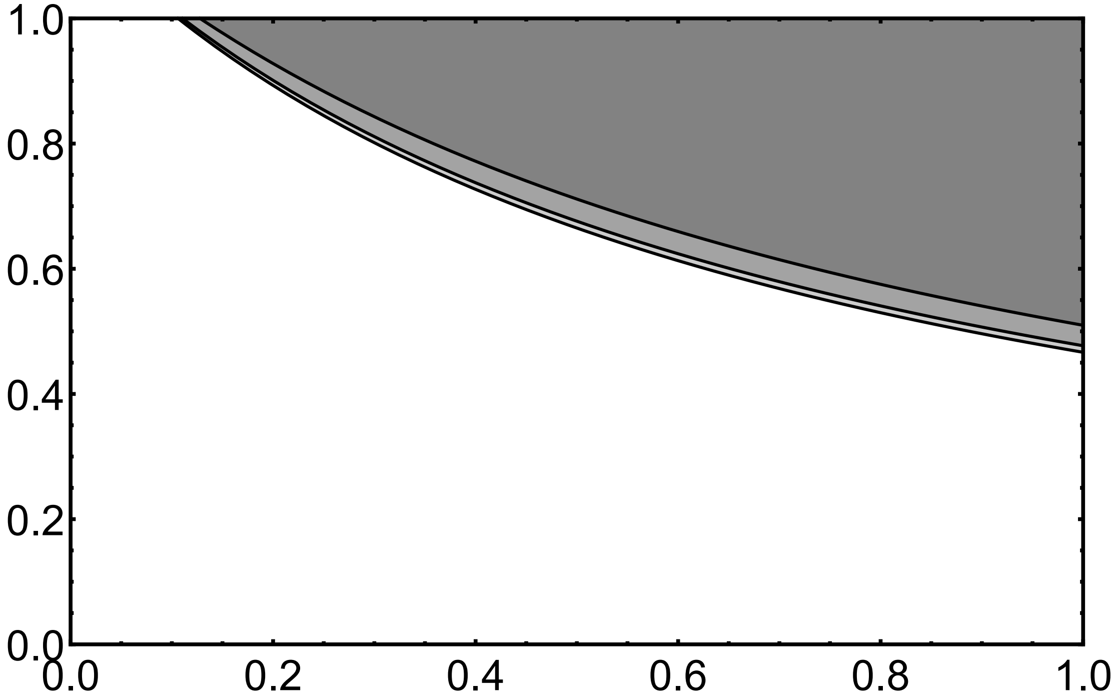

This plot shows the influence of the value of d on the size of the region in which helping is favored over breeding, with all parameters drawn from the western bluebird population. The x-axis is s and the y-axis is g. The top line represents d=1, the bottom line d=6. The larger d is, the larger the region in which helping is favored over breeding, but the effect size is small.

Figure 2 e) ratio of offspring number produced by a group with a helper to offspring number produced by a pair (o_h/o_b)

Note: the ratio in the western bluebirds, with $o_b = 2.89$ and $o_h = 3.56$, is 1.23183 to 1.

1. Ratio is 1.1:1

```
wh1ratio11 = wh1 /. ro → 0.5 /. ob → 1 /. oh → 1.1 /. oe → 1.87 /. d → 3
wb1ratio11 = wb1 /. ro → 0.5 /. ob → 1 /. oh → 1.1 /. oe → 1.87 /. d → 3
```

$0.025\,(0.25\,g\,s + 0.166667\,(4 - 1.\,g\,s)\,(3. - 0.5\,g\,s))$

$0.5\,(1 - 1.87\,(-1 + g) - g\,s)$

```
Reduce[wh1ratio11 > wb1ratio11 && 0 < s < 1 && 0 < g < 1]
```

Reduce::ratnz : Reduce was unable to solve the system with inexact coefficients.
The answer was obtained by solving a corresponding exact system and numericizing the result. >>

$$0.923379 < s < 1.\ \&\&\ -\frac{0.1\,(2244. + 1165.\,s)}{s^2} + 2.86145\times10^{-9}\sqrt{\left(\frac{1}{s^4}\left(6.14998\times10^{21} + 6.38567\times10^{21}\,s + 1.73879\times10^{21}\,s^2\right)\right)} < g < 1.$$

$$\text{g11plot} = -\frac{1}{s^2}\,0.09999999999999992\text{`}\,(2244.\text{`} + 1165.\text{`}\,s) + 2.8614497735611808\text{`*\^{}-9}\sqrt{\left(\frac{1}{s^4}\left(6.14997642120757\text{`*\^{}21} + 6.385670704729786\text{`*\^{}21}\,s + 1.7387924901649643\text{`*\^{}21}\,s^2\right)\right)};$$

```
Plot11 = Plot[g11plot, {s, 0.9233792734649543, 1}, PlotRange → {{0, 1}, {0, 1}},
  Filling → Top, PlotStyle → Black, LabelStyle → Directive[Black, FontSize → 22],
  ImageSize → Large, Frame → True, FrameStyle → Directive[Thick, Black]]
```

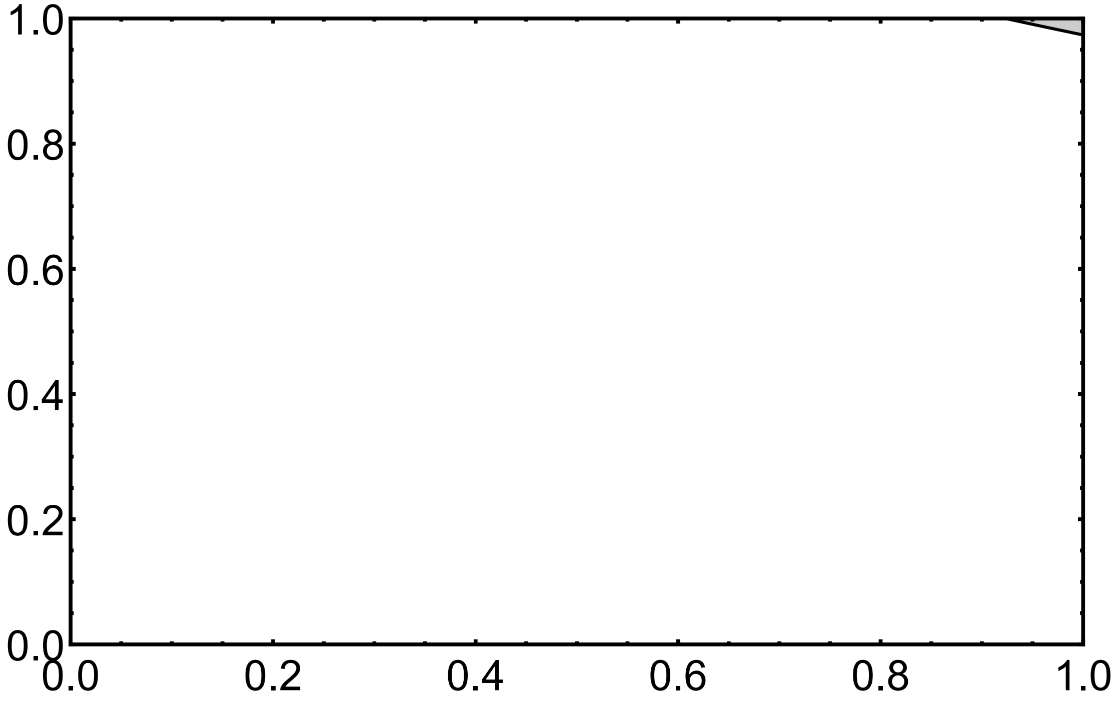

2. Ratio is 1.2:1

$wh1ratio12 = w_{h1}$ /. $r_o \to 0.5$ /. $o_b \to 1$ /. $o_h \to 1.2$ /. $o_e \to 1.87$ /. $d \to 3$

$wb1ratio12 = w_{b1}$ /. $r_o \to 0.5$ /. $o_b \to 1$ /. $o_h \to 1.2$ /. $o_e \to 1.87$ /. $d \to 3$

$0.05\,(0.25\,g\,s + 0.166667\,(4 - 1.\,g\,s)\,(3. - 0.5\,g\,s))$

$0.5\,(1 - 1.87\,(-1 + g) - g\,s)$

```
Reduce[wh1ratio12 > wb1ratio12 && 0 < s < 1 && 0 < g < 1]
```

Reduce::ratnz : Reduce was unable to solve the system with inexact coefficients.
The answer was obtained by solving a corresponding exact system and numericizing the result. ≫

$$0.843265 < s < 1.\ \&\&\ -\frac{0.1\,(1122. + 565.\,s)}{s^2} + 1.49012\times 10^{-9}\sqrt{\left(\frac{1}{s^4}\left(5.66951\times 10^{21} + 5.70993\times 10^{21}\,s + 1.58196\times 10^{21}\,s^2\right)\right)} < g < 1.$$

$$g12plot = -\frac{1}{s^2}\,0.10000000000000002`\,(1122.` + 565.`\,s) + 1.4901161193847684`*\hat{}-9\sqrt{\left(\frac{1}{s^4}\left(5.669509513302661`*\hat{}21 + 5.709933823557938`*\hat{}21\,s + 1.581956923108292`*\hat{}21\,s^2\right)\right)};$$

```
Plot12 = Plot[g12plot, {s, 0.8432646437225525, 1}, PlotRange → {{0, 1}, {0, 1}},
  Filling → Top, PlotStyle → Black, LabelStyle → Directive[Black, FontSize → 22],
  ImageSize → Large, Frame → True, FrameStyle → Directive[Thick, Black]]
```

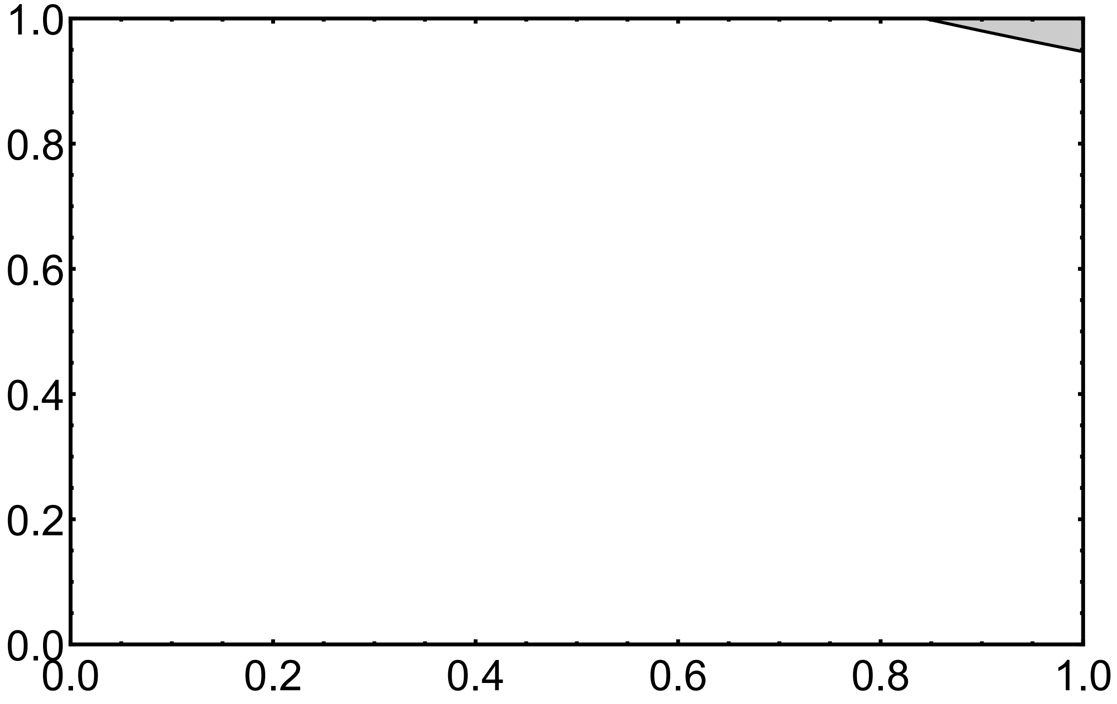

3. Ratio is 1.3:1

```
wh1ratio13 = wh1 /. ro → 0.5 /. ob → 1 /. oh → 1.3 /. oe → 1.87 /. d → 3
wb1ratio13 = wb1 /. ro → 0.5 /. ob → 1 /. oh → 1.3 /. oe → 1.87 /. d → 3
```

$0.075\ (0.25\ g\ s + 0.166667\ (4 - 1.\ g\ s)\ (3. - 0.5\ g\ s))$

$0.5\ (1 - 1.87\ (-1 + g) - g\ s)$

```
Reduce[wh1ratio13 > wb1ratio13 && 0 < s < 1 && 0 < g < 1]
```

Reduce::ratnz : Reduce was unable to solve the system with inexact coefficients.
The answer was obtained by solving a corresponding exact system and numericizing the result. >>

$$0.759227 < s < 1.\ \&\&\ \frac{0.1\ (-748. - 365.\ s)}{s^2} + 0.1\sqrt{\left(\frac{1}{s^4}\left(559504. + 546040.\ s + 153785.\ s^2\right)\right)} < g < 1.$$

$$\text{g13plot} = \frac{0.1\grave{}\ (-748.\grave{} - 365.\grave{}\ s)}{s^2} + 0.1\grave{}\sqrt{\left(\frac{1}{s^4}\left(559504.\grave{} + 546040.\grave{}\ s + 153785.\grave{}\ s^2\right)\right)};$$

```
Plot13 = Plot[g13plot, {s, 0.7592270451226639, 1}, PlotRange → {{0, 1}, {0, 1}},
  Filling → Top, PlotStyle → Black, LabelStyle → Directive[Black, FontSize → 22],
  ImageSize → Large, Frame → True, FrameStyle → Directive[Thick, Black]]
```

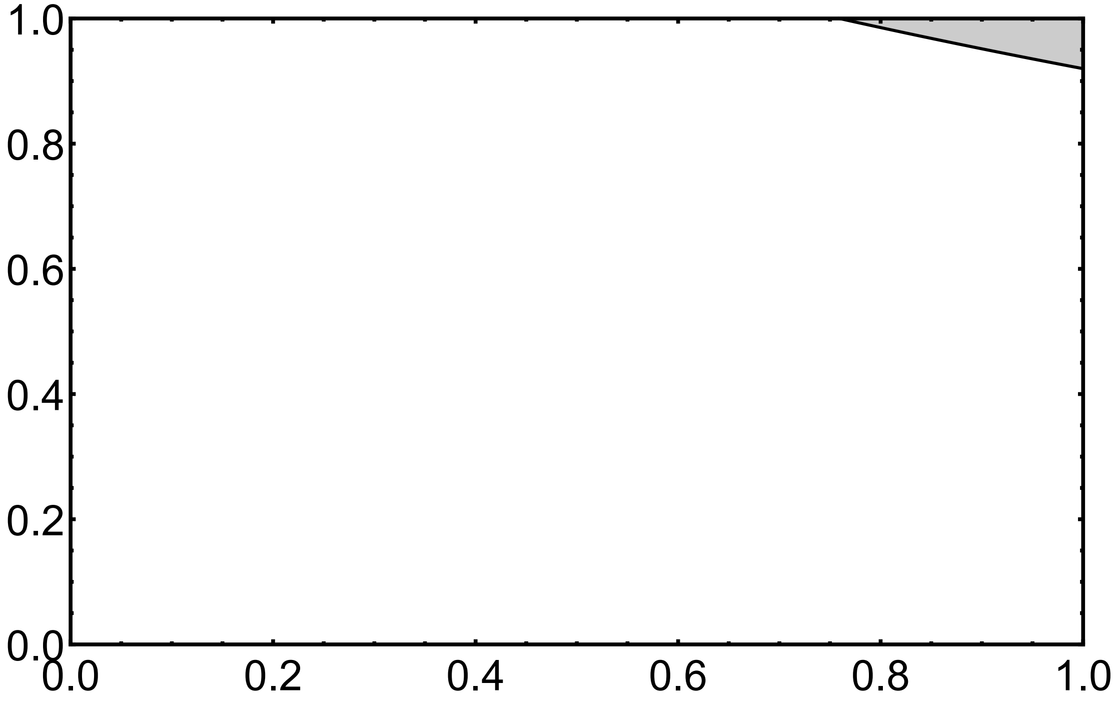

4. Ratio is 1.4:1

```
wh1oratio14 = wh1 /. ro → 0.5 /. ob → 1 /. oh → 1.4 /. oe → 1.87 /. d → 3
wb1oratio14 = wb1 /. ro → 0.5 /. ob → 1 /. oh → 1.4 /. oe → 1.87 /. d → 3
```

$0.1\,(0.25\,g\,s + 0.166667\,(4 - 1.\,g\,s)\,(3. - 0.5\,g\,s))$

$0.5\,(1 - 1.87\,(-1 + g) - g\,s)$

```
Reduce[wh1oratio14 > wb1oratio14 && 0 < s < 1 && 0 < g < 1]
```

Reduce::ratnz : Reduce was unable to solve the system with inexact coefficients.
The answer was obtained by solving a corresponding exact system and numericizing the result. ≫

$$0.670756 < s < 1.\ \&\&\ -\frac{0.1\,(561. + 265.\,s)}{s^2} + 1.49012 \times 10^{-9} \sqrt{\left(\frac{1}{s^4}\left(1.41738 \times 10^{21} + 1.33906 \times 10^{21}\,s + 3.83009 \times 10^{20}\,s^2\right)\right)} < g < 1.$$

$$\texttt{g14plot} = -\frac{1}{s^2}\,0.10000000000000002\text{`}\,(561.\text{`} + 265.\text{`}\,s) + 1.4901161193847684\text{`*\^{}-9}\sqrt{\left(\frac{1}{s^4}\left(1.4173773783256652\text{`*\^{}21} + 1.339055277206065\text{`*\^{}21}\,s + 3.830086303097225\text{`*\^{}20}\,s^2\right)\right)};$$

```
Plot14 = Plot[g14plot, {s, 0.6707563383870486, 1}, PlotRange → {{0, 1}, {0, 1}},
  Filling → Top, PlotStyle → Black, LabelStyle → Directive[Black, FontSize → 22],
  ImageSize → Large, Frame → True, FrameStyle → Directive[Thick, Black]]
```

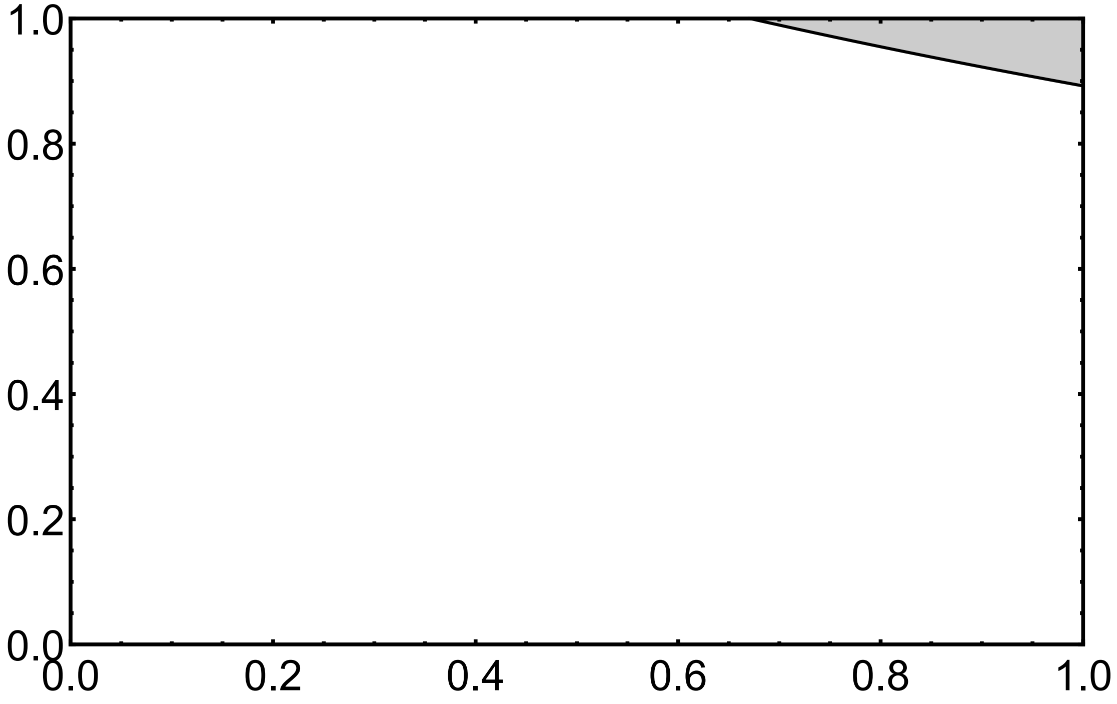

5. Ratio is 1.5:1

wh1oratio15 = w_{h1} **/.** r_o **→ 0.5 /.** o_b **→ 1 /.** o_h **→ 1.5 /.** o_e **→ 1.87 /. d → 3**

wb1oratio15 = w_{b1} **/.** r_o **→ 0.5 /.** o_b **→ 1 /.** o_h **→ 1.5 /.** o_e **→ 1.87 /. d → 3**

$0.125\ (0.25\ g\ s + 0.166667\ (4 - 1.\ g\ s)\ (3. - 0.5\ g\ s))$

$0.5\ (1 - 1.87\ (-1 + g) - g\ s)$

Reduce[wh1oratio15 > wb1oratio15 && 0 < s < 1 && 0 < g < 1]

Reduce::ratnz : Reduce was unable to solve the system with inexact coefficients.
The answer was obtained by solving a corresponding exact system and numericizing the result. >>

$$0.577239 < s < 1.\ \&\&\ \frac{0.02\ (-2244. - 1025.\ s)}{s^2} + 0.02\ \sqrt{\left(\frac{1}{s^4}\left(5.03554\times 10^6 + 4.6002\times 10^6\ s + 1.33503\times 10^6\ s^2\right)\right)} < g < 1.$$

$$\textbf{g15plot} = \frac{0.02`\ (-2244.` - 1025.`\ s)}{s^2} + 0.02`\ \sqrt{\left(\frac{1}{s^4}\left(5.035536`*\string^6 + 4.6002`*\string^6\ s + 1.335025`*\string^6\ s^2\right)\right)};$$

```
Plot15 = Plot[g15plot, {s, 0.577238908357991, 1}, PlotRange → {{0, 1}, {0, 1}},
  Filling → Top, PlotStyle → Black, LabelStyle → Directive[Black, FontSize → 22],
  ImageSize → Large, Frame → True, FrameStyle → Directive[Thick, Black]]
```

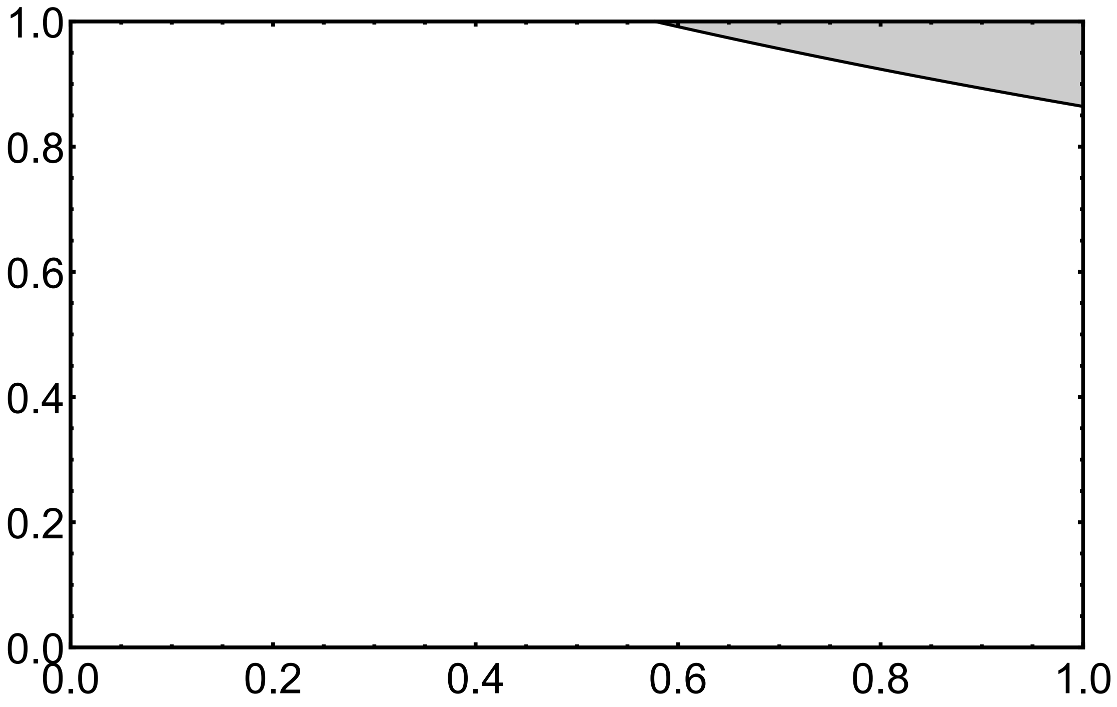

6. Ratio is 1.6:1

$wh1ratio16 = w_{h1} \;/.\; r_o \to 0.5 \;/.\; o_b \to 1 \;/.\; o_h \to 1.6 \;/.\; o_e \to 1.87 \;/.\; d \to 3$

$wb1ratio16 = w_{b1} \;/.\; r_o \to 0.5 \;/.\; o_b \to 1 \;/.\; o_h \to 1.6 \;/.\; o_e \to 1.87 \;/.\; d \to 3$

$0.15\,(0.25\,g\,s + 0.166667\,(4 - 1.\,g\,s)\,(3. - 0.5\,g\,s))$

$0.5\,(1 - 1.87\,(-1 + g) - g\,s)$

```
Reduce[wh1ratio16 > wb1ratio16 && 0 < s < 1 && 0 < g < 1]
```

Reduce::ratnz : Reduce was unable to solve the system with inexact coefficients.
The answer was obtained by solving a corresponding exact system and numericizing the result. ≫

$0.477927 < s < 1. \;\&\&$

$$-\frac{1.1\,(34. + 15.\,s)}{s^2} + 0.1\,\sqrt{\left(\frac{1}{s^4}\left(139\,876. + 123\,420.\,s + 36\,305.\,s^2\right)\right)} < g < 1.$$

$$g16plot = -\frac{1.1`\,(34.` + 15.`\,s)}{s^2} + 0.1`\,\sqrt{\left(\frac{1}{s^4}\left(139876.` + 123420.`\,s + 36305.`\,s^2\right)\right)};$$

```
Plot16 = Plot[g16plot, {s, 0.47792684634964644, 1}, PlotRange → {{0, 1}, {0, 1}},
  Filling → Top, PlotStyle → Black, LabelStyle → Directive[Black, FontSize → 22],
  ImageSize → Large, Frame → True, FrameStyle → Directive[Thick, Black]]
```

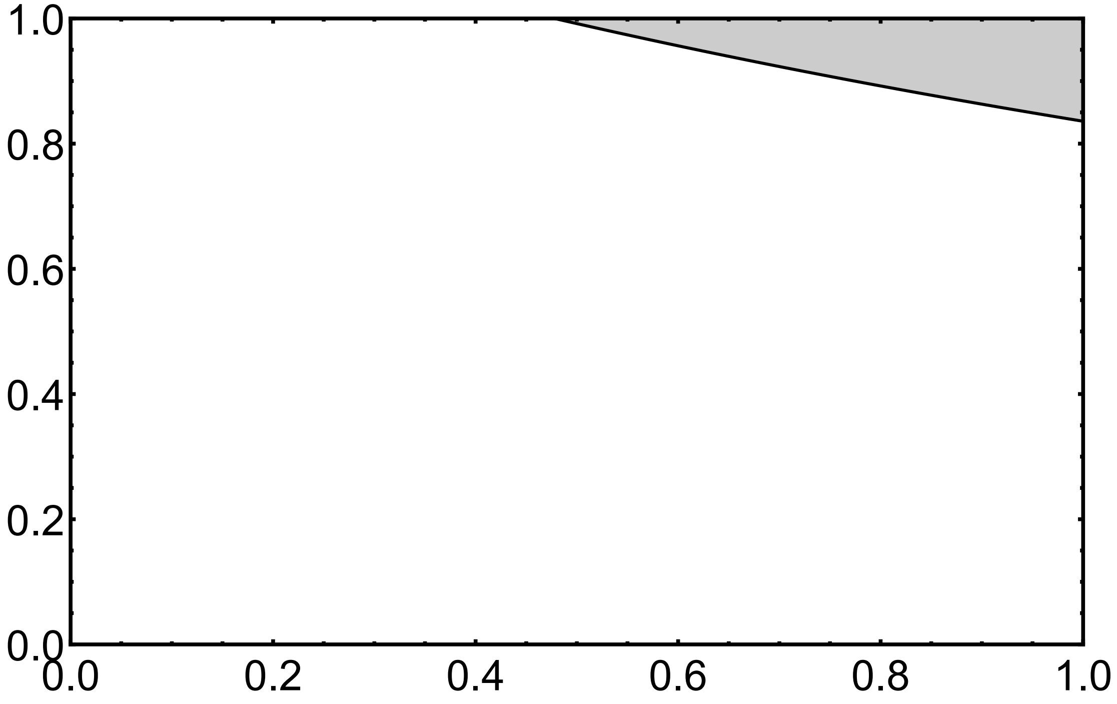

7. Ratio is 1.7:1

$wh1ratio17 = w_{h1}$ /. $r_o \to 0.5$ /. $o_b \to 1$ /. $o_h \to 1.7$ /. $o_e \to 1.87$ /. $d \to 3$

$wb1ratio17 = w_{b1}$ /. $r_o \to 0.5$ /. $o_b \to 1$ /. $o_h \to 1.7$ /. $o_e \to 1.87$ /. $d \to 3$

$0.175\ (0.25\ g\ s + 0.166667\ (4 - 1.\ g\ s)\ (3. - 0.5\ g\ s))$

$0.5\ (1 - 1.87\ (-1 + g) - g\ s)$

```
Reduce[wh1ratio17 > wb1ratio17 && 0 < s < 1 && 0 < g < 1]
```

Reduce::ratnz : Reduce was unable to solve the system with inexact coefficients.
The answer was obtained by solving a corresponding exact system and numericizing the result. ≫

$$0.371895 < s < 1.\ \&\&\ \frac{0.0142857\ (-2244. - 955.\ s)}{s^2} + 0.0142857\sqrt{\left(\frac{1}{s^4}\left(5.03554\times10^6 + 4.28604\times10^6\ s + 1.27659\times10^6\ s^2\right)\right)} < g < 1.$$

$$g17plot = \frac{1}{s^2}\ 0.014285714285714285\text{`}\ (-2244.\text{`} - 955.\text{`}\ s) + 0.014285714285714285\text{`}\sqrt{\left(\frac{1}{s^4}\left(5.035536\text{`}*\text{^}6 + 4.28604\text{`}*\text{^}6\ s + 1.276585\text{`}*\text{^}6\ s^2\right)\right)};$$

```
Plot17 = Plot[g17plot, {s, 0.37189456047754277, 1}, PlotRange → {{0, 1}, {0, 1}},
  Filling → Top, PlotStyle → Black, LabelStyle → Directive[Black, FontSize → 22],
  ImageSize → Large, Frame → True, FrameStyle → Directive[Thick, Black]]
```

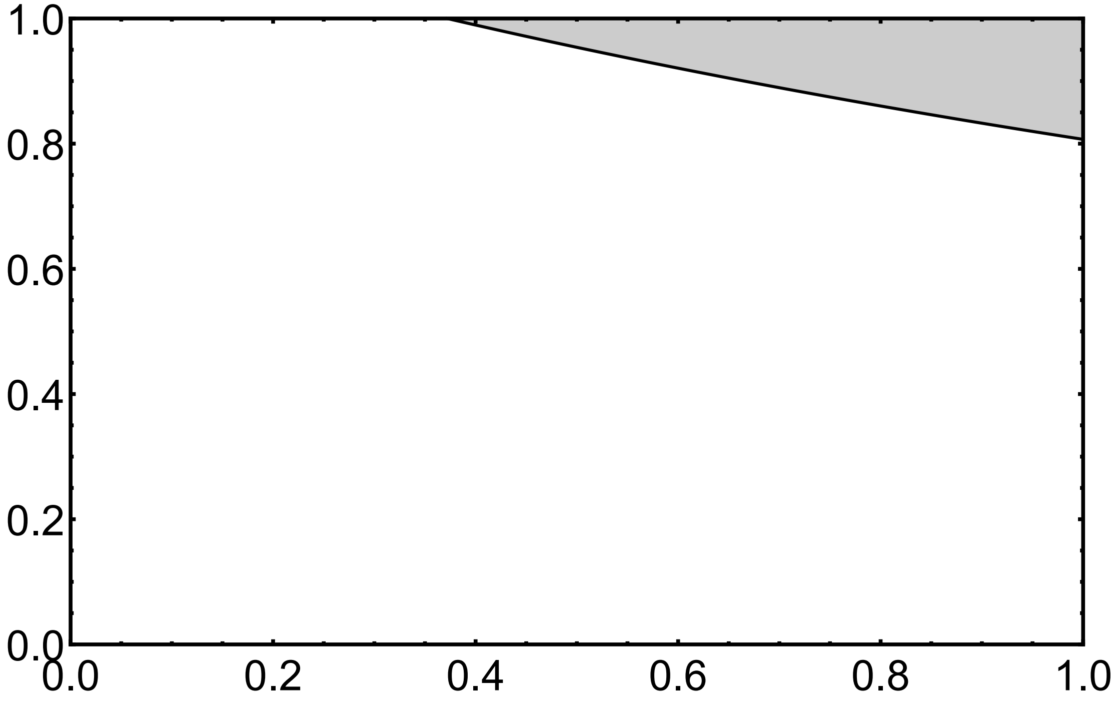

8. Ratio is 1.8:1

```
wh1ratio18 = wh1 /. ro → 0.5 /. ob → 1 /. oh → 1.8 /. oe → 1.87 /. d → 3
wb1ratio18 = wb1 /. ro → 0.5 /. ob → 1 /. oh → 1.8 /. oe → 1.87 /. d → 3
```

$0.2\ (0.25\ g\ s + 0.166667\ (4 - 1.\ g\ s)\ (3. - 0.5\ g\ s))$

$0.5\ (1 - 1.87\ (-1 + g) - g\ s)$

```
Reduce[wh1ratio18 > wb1ratio18 && 0 < s < 1 && 0 < g < 1]
```

Reduce::ratnz : Reduce was unable to solve the system with inexact coefficients.
The answer was obtained by solving a corresponding exact system and numericizing the result. ≫

$$0.257976 < s < 1.\ \&\&\ \frac{0.05\ (-561. - 230.\ s)}{s^2} + 8.42937\times 10^{-9} \sqrt{\left(\frac{1}{s^4}\left(1.10733\times 10^{19} + 9.07968\times 10^{18}\ s + 2.73523\times 10^{18}\ s^2\right)\right)} < g < 1.$$

```
g18plot =
  0.05` (-561.` - 230.` s) / s^2 + 8.42936970219977`*^-9 Sqrt[(1/s^4 (1.107326076811422`*^19 +
        9.079679061198825`*^18 s + 2.7352330861721953`*^18 s^2))];
```

```
Plot18 = Plot[g18plot, {s, 0.25797601630484807, 1}, PlotRange → {{0, 1}, {0, 1}},
  Filling → Top, PlotStyle → Black, LabelStyle → Directive[Black, FontSize → 22],
  ImageSize → Large, Frame → True, FrameStyle → Directive[Thick, Black]]
```

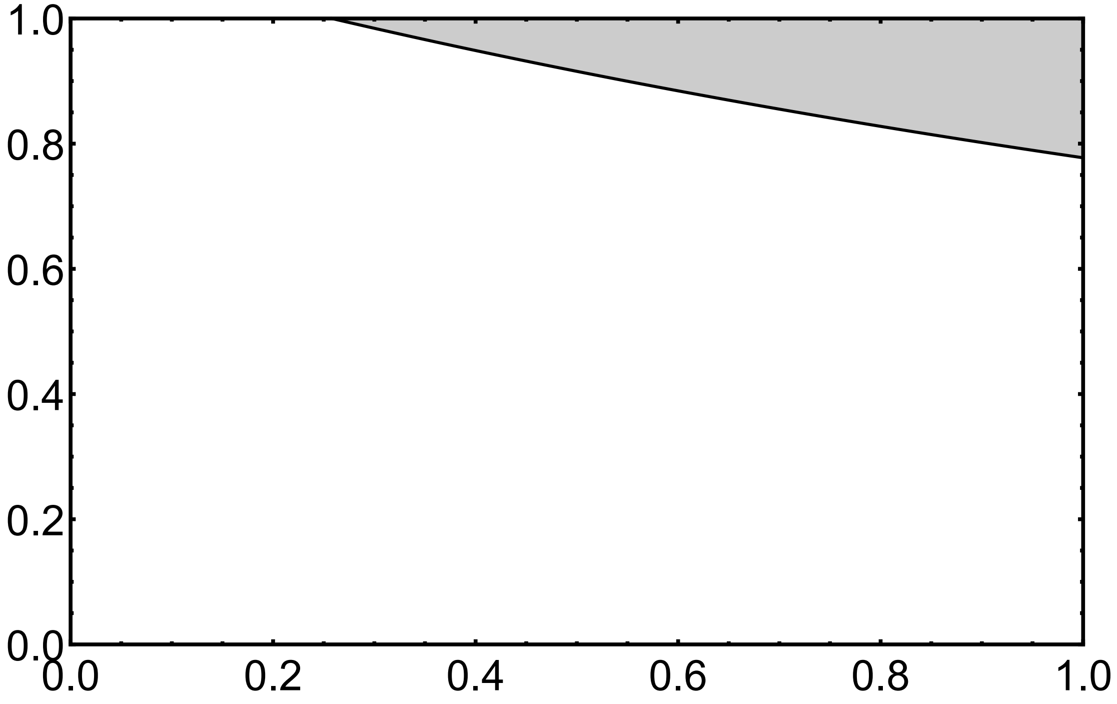

9. Ratio is 1.9:1

```
wh1oratio19 = wh1 /. ro → 0.5 /. σb → 1 /. σh → 1.9 /. σe → 1.87 /. d → 3
wb1oratio19 = wb1 /. ro → 0.5 /. σb → 1 /. σh → 1.9 /. σe → 1.87 /. d → 3
```

0.225 (0.25 g s + 0.166667 (4 - 1. g s) (3. - 0.5 g s))

0.5 (1 - 1.87 (-1 + g) - g s)

```
Reduce[wh1oratio19 > wb1oratio19 && 0 < s < 1 && 0 < g < 1]
```

Reduce::ratnz : Reduce was unable to solve the system with inexact coefficients.

The answer was obtained by solving a corresponding exact system and numericizing the result. >>

$$0.134671 < s < 1. \&\& -\frac{0.0333333\,(748. + 295.\,s)}{s^2} + 8.60319 \times 10^{-10} \sqrt{\left(\frac{1}{s^4}\left(8.39927 \times 10^{20} + 6.6251 \times 10^{20}\,s + 2.01619 \times 10^{20}\,s^2\right)\right)} < g < 1.$$

$$\text{g19plot} = -\frac{1}{s^2}\,0.03333333333333334\grave{}\,(748.\grave{} + 295.\grave{}\,s) + 8.603189426506348\grave{}*\hat{}{-10}\sqrt{\left(\frac{1}{s^4}\left(8.399273353040232\grave{}*\hat{}20 + 6.625095291836548\grave{}*\hat{}20\,s + 2.0161864931797955\grave{}*\hat{}20\,s^2\right)\right)};$$

```
Plot19 = Plot[g19plot, {s, 0.13467103620378573, 1}, PlotRange → {{0, 1}, {0, 1}},
  Filling → Top, PlotStyle → Black, LabelStyle → Directive[Black, FontSize → 22],
  ImageSize → Large, Frame → True, FrameStyle → Directive[Thick, Black]]
```

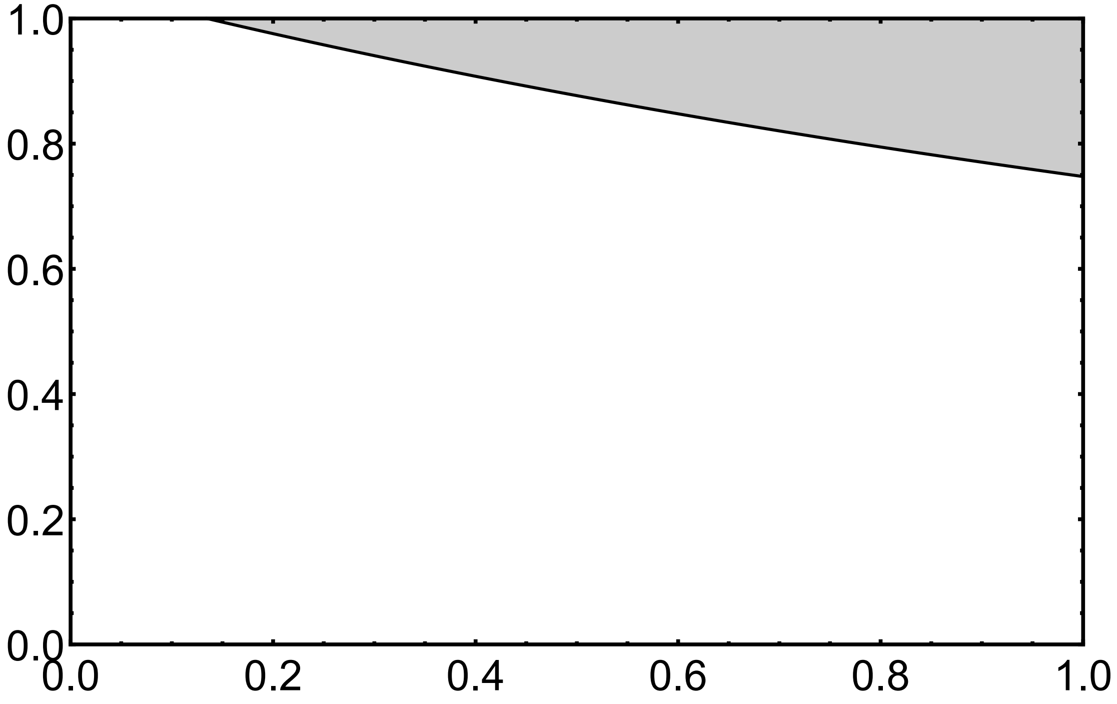

10. Ratio is 2:1

```
wh1oratio20 = wh1 /. ro → 0.5 /. ob → 1 /. oh → 2 /. oe → 1.87 /. d → 3
wb1oratio20 = wb1 /. ro → 0.5 /. ob → 1 /. oh → 2 /. oe → 1.87 /. d → 3
```

$\frac{1}{4}$ (0.25 g s + 0.166667 (4 − 1. g s) (3. − 0.5 g s))

0.5 (1 − 1.87 (−1 + g) − g s)

```
Reduce[wh1oratio20 > wb1oratio20 && 0 < s < 1 && 0 < g < 1]
```

Reduce::ratnz : Reduce was unable to solve the system with inexact coefficients.
The answer was obtained by solving a corresponding exact system and numericizing the result. >>

$$0 < s < 1. \;\&\&\; -\frac{0.34\,(66. + 25.\,s)}{s^2} + 0.0824621\sqrt{\left(\frac{1}{s^4}\left(74\,052. + 56\,100.\,s + 17\,225.\,s^2\right)\right)} < g < 1.$$

$$\text{g20plot} = -\frac{0.34`\,(66.` + 25.`\,s)}{s^2} + 0.08246211251235322`\sqrt{\left(\frac{1}{s^4}\left(74052.` + 56100.`\,s + 17225.`\,s^2\right)\right)};$$

```
Plot20 = Plot[g20plot, {s, 0, 1}, PlotRange → {{0, 1}, {0, 1}}, Filling → Top,
  PlotStyle → Black, LabelStyle → Directive[Black, FontSize → 22],
  ImageSize → Large, Frame → True, FrameStyle → Directive[Thick, Black]]
```

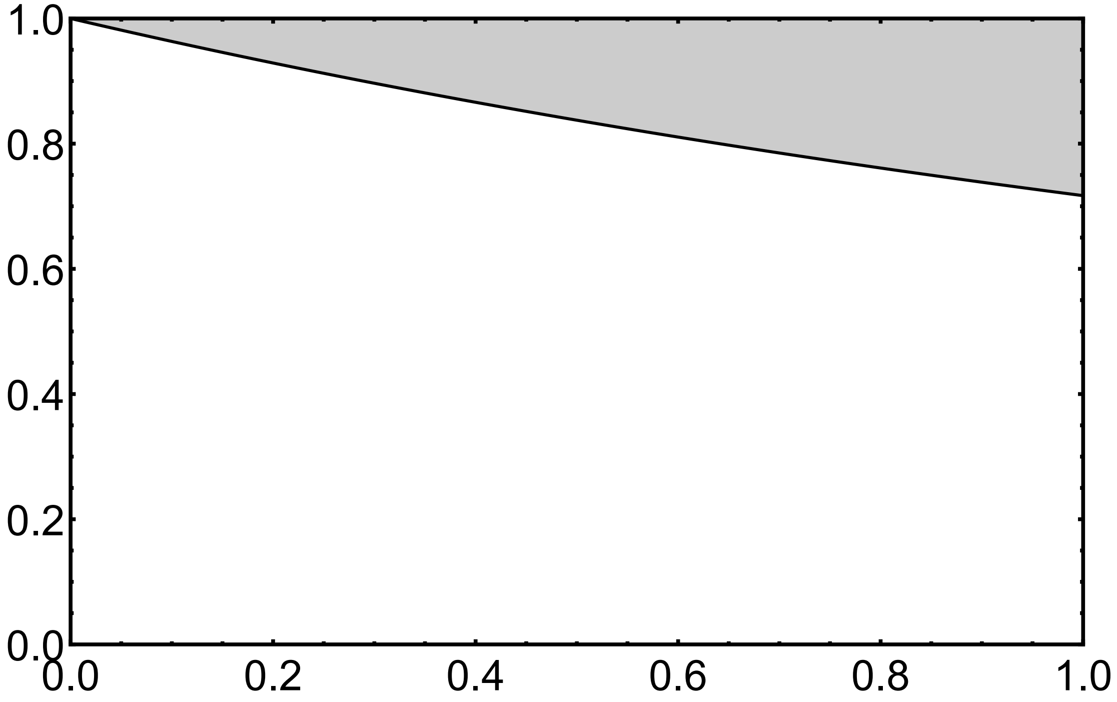

Overlay these plots:

```
PlotAllohob =
 Show[Plot11, Plot12, Plot13, Plot14, Plot15, Plot16, Plot17, Plot18, Plot19, Plot20]
```

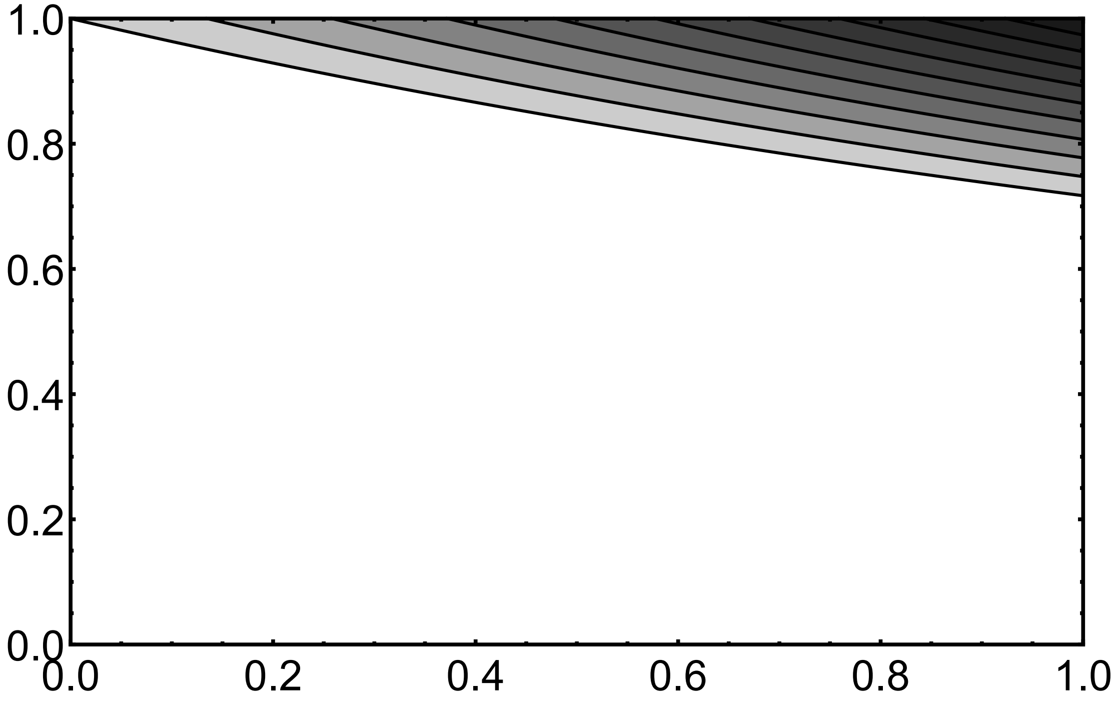

This plot shows the influence of the ratio of o_h to o_b on the size of the region in which helping is favored over breeding, with all parameters except the o_h and o_b values drawn from the western bluebird population. The x-axis is s and the y-axis is g. The top line represents the o_h:o_b ratio 1.1:1, the bottom line 2:1.
