# Supplementary material for: Effects of load-lightening and delayed extrapair benefits on the fitness consequences of helping behavior
Source: Behav Ecol. 2016 Feb 17;27(4):1078–86. doi: 10.1093/beheco/arw018 (PMC6191074; doi:10.1093/beheco/arw018)
Supplement: Supplementary Data [file supp_27_4_1078__index.html]

Effects of load-lightening and delayed extrapair benefits on the fitness consequences of helping behavior — Effects of load-lightening and delayed extrapair benefits on the fitness consequences of helping behavior — Supplementary Data 

# Effects of load-lightening and delayed extrapair benefits on the fitness consequences of helping behavior

## Supplementary Data

Data files

- Supplementary Data - Supplementary Data
- Supplementary Data - Supplementary Data
